# Epigenetic suppression of liver X receptor β in anterior cingulate cortex by HDAC5 drives CFA-induced chronic inflammatory pain

**DOI:** 10.1186/s12974-019-1507-3

**Published:** 2019-06-29

**Authors:** Yu-Jiao Li, Kun Zhang, Ting Sun, Jian Wang, Yan-Yan Guo, Le Yang, Qi Yang, Yan-Jiao Li, Shui-Bing Liu, Ming-Gao Zhao, Yu-Mei Wu

**Affiliations:** 1 Department of Pharmacy, Precision Pharmacy & Drug Development Center, The Second Affiliated Hospital, Fourth Military Medical University, Xi’an, 710038 Shaanxi Province People’s Republic of China; 2 Department of Pharmacology, School of Pharmacy, Fourth Military Medical University, Xi’an, 710032 Shaanxi Province People’s Republic of China; 3 Department of Ambulatorium, 94750 Army Hospital, Liancheng, 366200 FuJian Province People’s Republic of China; 4 Department of Acupuncture and Moxibustion, Xi’an Hospital of Traditional Chinese Medicine, Xi’an, 710021 Shaanxi Province People’s Republic of China

**Keywords:** Chronic inflammatory pain, Anterior cingulate cortex, Liver X receptors, Histone modification, Neuroinflammation, Neurotransmission

## Abstract

**Background:**

Liver X receptors (LXRs), including LXRα and LXRβ, are key regulators of transcriptional programs for both cholesterol homeostasis and inflammation in the brain. Here, the modes of action of LXRs and the epigenetic mechanisms regulating LXRβ expression in anterior cingulate cortex (ACC) of chronic inflammatory pain (CIP) are investigated.

**Methods:**

The deficit of LXR isoform and analgesic effect of LXR activation by GW3965 were evaluated using the mouse model of CIP induced by hindpaw injection of complete Freund’s adjuvant (CFA). The mechanisms involved in GW-mediated analgesic effects were analyzed with immunohistochemical methods, ELISA, co-immunoprecipitation (Co-IP), Western blot, and electrophysiological recording. The epigenetic regulation of LXRβ expression was investigated by chromatin immunoprecipitation, quantitative real-time PCR, and sequencing.

**Results:**

We revealed that CFA insult led to LXRβ reduction in ACC, which was associated with upregulated expression of histone deacetylase 5 (HDAC5), and knockdown of LXRβ by shRNA led to thermal hyperalgesia. Co-IP showed that LXRβ interacted with NF-κB p65 physically. LXRβ activation by GW3965 exerted analgesic effects by inhibiting the nuclear translocation of NF-κB, reducing the phosphorylation of mitogen-activated protein kinases (MAPKs) in ACC, and decreasing the promoted input-output and enhanced mEPSC frequency in ACC neurons after CFA exposure. In vitro experiments confirmed that HDAC5 triggered histone deacetylation on the promoter region of *Lxrβ*, resulting in downregulation of *Lxrβ* transcription.

**Conclusion:**

These findings highlight an epigenetic mechanism underlying LXRβ deficits linked to CIP, and LXRβ activation may represent a potential novel target for the treatment of CIP with an alteration in inflammation responses and synaptic transmission in ACC.

**Electronic supplementary material:**

The online version of this article (10.1186/s12974-019-1507-3) contains supplementary material, which is available to authorized users.

## Background

Chronic pain is defined as a neuroepigenetic disorder caused by persistent tissue inflammation or nerve injury under various pathological conditions, which is accompanied by lasting, multifaceted maladaptations ranging from gene modulation to synaptic dysfunction and emotional disorders [1, 2]. Inflammation, tissue injury, and/or nerve injury-induced changes of gene expression in sensory neurons from the dorsal root ganglion, dorsal horn of spinal cord, and pain-associated brain regions are thought to participate in chronic pain genesis; however, how these changes occur is still elusive and efforts have been made to look for the genetic mechanisms involved in the regulation of gene expression. Epigenetic modifications, including DNA methylation, histone modifications, and microRNAs, strongly govern gene expression, potentially for long periods over years or even generations, and have been associated with the induction of pain hypersensitivity under chronic pain conditions [3].

Studies demonstrated that many genes undergo expression changes at mRNA and protein levels in tissues or cells of pain circuitry during the development or maintenance of persistent pain [3]. Liver X receptors (LXRs), including LXRα/NR1h3 and LXRβ/NR1h2, belong to the nuclear receptor superfamily of transcription factors [4]. LXRs have emerged as important regulators controlling cellular and whole-body cholesterol homeostasis [5]. Given the complexity of metabolic regulation by LXRs, it has been difficult to define their roles in normal function or disease states. LXRα is expressed predominantly in organs of lipid metabolism, whereas LXRβ is expressed ubiquitously in most of the physical system and plays crucial roles in the immune system and central nervous system (CNS). LXRα knockout did not cause fundamental developmental defects in mice, whereas LXRβ deletion led to impaired cerebral cortex lamination [6], neurodegeneration in substantia nigra [7], anxiety, and impaired behavior response [8]. Furthermore, LXRβ was also expressed in the spinal cord, and male LXRβ^-/-^ mice suffered from adult-onset motor neuron degeneration [9], whereas LXRβ activation by T0901317 protected the spinal cord from injury [10]. Both T0901317 and GW3965 (GW) [11] are synthetic ligands for LXRs, and GW is an LXR full agonist for both LXRα and LXRβ isoforms and can readily cross the blood-brain barrier to exert its specific actions in the brain [12]. Notably, the role of LXRs in chronic pain remains largely unknown.

Central sensitization plays a key role in the process of chronic pain, which is associated with the activation of a distributed group of structures, including the somatosensory cortex, the insular cortex, and the anterior cingulate cortex (ACC) [13, 14]. ACC attracts more and more attention in pain research which plays key roles in pain modulation, learning, memory, and attention [15, 16]. Our previous work revealed a significant enhancement in the probability of neurotransmitter release in ACC synapses from mice with chronic pain [17]. More and more evidence linked central sensitization to abnormal gene expression within the cells processing nociceptive signaling in CNS [3, 18].

In this study, we investigated whether dysfunction of LXRβ led to hyperalgesia and the epigenetic regulation mechanisms involved. Here, we demonstrated the downregulation of LXRβ not LXRα in ACC of chronic inflammatory pain (CIP) mice, and knockdown of LXRβ by short hairpin RNA (shRNA) led to thermal hyperalgesia, while activation of LXRβ by GW exerted analgesic effects through both anti-inflammation and correction in synaptic transmission in ACC. Chromatin immunoprecipitation (ChIP) analysis showed that histone deacetylase 5 (HDAC5) triggered histone deacetylation on the promoter region of *Lxrβ*, resulting in the reduction of *Lxrβ* transcription in cultured cortical neurons. Taken together, these findings highlight an epigenetic mechanism underlying LXRβ deficits linked to CIP, revealing potentially targetable receptor for clinical intervention in CIP.

## Materials and methods

### Animals

Adult male C57BL/6 mice aged 6–8 weeks were purchased from the Fourth Military Medical University Experimental Animal Center (Xi’an, China). Animals were housed in groups of five under standard laboratory conditions (24 ± 2 °C, 12-h light/dark cycle, food and water ad libitum). All behavioral tests were performed during the light period on the designated day of experiment. All experimental procedures were approved by the Fourth Military Medical University Animal Care and Use Committee. Every effort was made to minimize the number of animals used and their suffering.

### Experimental designs and GW3965 treatment

The model of CIP was established by hindpaw CFA injection according to previous studies [19–21]. Either GW (1 and 10 mg/kg, Selleckchem, Shanghai, China) or 0.9% saline (vehicle, 0.2 ml) was administered intraperitoneally (*i.p*) immediately after CFA insult (10 μl, 50% in saline) for another consecutive 14 days. All the samples for Western blot and immunohistochemistry were collected from the ipsilateral ACC of CFA injection into the hindpaw.

### Nociceptive behavioral tests

Mechanical allodynia was assessed by von Frey filaments [22], and thermal hyperalgesia was evaluated via a plantar analgesia device (BME410A, China), which is measured as paw withdrawal threshold according to previous research [23].

### shRNA lentivirus construction and transfection

To knock down endogenous LXRs, shRNA-coding plasmids against LXRα (NM_013839.4) and LXRβ (NM_009473.3) were designed according to validated shRNA sequences [24]. Lentivirus (pGLV-U6-GFP)-encoding shRNAs for LXRα and LXRβ were prepared by GenePharma (Shanghai, China). The sequence of shRNA for LXRα (shLXRα) was 5′-TGCCTGATGTTTCTCCTGAT-3′, and that of shRNA for LXRβ (shLXRβ) was 5′-GGATTCAGAAGCAGCAACAT-3′. Negative shRNA for LXRs (shNC) served as a lentivirus infection control.

### Stereotaxic surgery and microinjections

Mice were anesthetized with a mixture of ketamine (30 mg/ml) and xylazine (3 mg/ml) and were mounted on a stereotaxic apparatus (RWD68001, Shenzhen Ruiwode Life Science, China). An attenuated glass electrode (approximately 10 μm in diameter) was implanted bilaterally into the ACC (+ 1.0 mm anteroposterior, ± 0.3 mm lateral to the midline, and − 1.5 mm dorsoventral). The mice received lentiviral shLXRα or shLXRβ (2 μl, 1 × 10^9^ gc/ml) bilaterally at 0.25 μl/min driven by an infusion pump (Harvard Apparatus, MA). shNC served as an infection control. The CIP model was set up 14 days after shRNA infection, and mice were treated as before.

### Locomotor activity tests

Behavioral tests were carried out 2 weeks after lentivirus injection. Locomotor activity was conducted using the open-field and rotarod test as described previously [25].

### Western blot analysis

Samples from mice ACC were collected. Total proteins were lysed by M-PER Protein Extraction Buffer, and nuclear proteins of ACC were extracted with nuclear extraction kit (Thermo) according to the manufacturer’s instructions. Protein concentrations were determined using a BCA Kit. Equal amounts of protein aliquots were used for Western blot analysis to check the expression levels of LXRα, LXRβ, p50, p65, HDAC2, HDAC5, Iba-1 (Abcam), phosphorylated IκBα (p-IκBα, Bioworld Technology), phosphorylated extracellular regulated protein kinases (p-ERKs), ERK, phosphorylated p38 (p-p38), p38, phosphorylated c-Jun N-terminal kinase (p-JNK), JNK, GFAP (Cell Signaling Technology), AcH3 and AcH4 (Millipore), Histone H3, Histone H4, β-tubulin III (Proteintech), and β-actin (Sigma-Aldrich) served as a loading control.

### Immunohistochemistry

Frozen sections were fixed in acetone for 10–15 min and incubated in 3% H_2_O_2_ in PBS for 15 min to quench endogenous peroxidase at room temperature. To block nonspecific binding, sections were blocked in 3% BSA for 10 min, after which a biotin blocking system (Dako) was used to block endogenous biotin. Sections were then incubated with rabbit anti-LXRβ in 1% BSA overnight at room temperature. BSA replaced the primary antibodies in the negative controls. After washing, sections were incubated with the corresponding secondary antibodies using a rabbit HRP-polymer kit (P0203; Beyotime) for 15–20 min at room temperature, followed by DAB as a chromogen. Total number of LXRβ-positive cells in ACC was counted using the optical fractionator method of unbiased stereology. Briefly, the optical dissector had a size of 20 μm × 20 μm in the *X*- and *Y*-axis from ACC of the section. Dissectors were positioned every 50 μm in the *X*- and *Y*-axis. The sampled region for each ACC subfield was demarcated, and DAB LXRβ-positive cells were counted. The estimated number of LXRβ positive cells in each field was divided by the area of the region of interest to obtain the cellular density expressed in cells per 0.04 mm^2^.

### Immunofluorescence

Animals were intracardially perfused with 0.9% saline and 4% PFA (wt/vol). Floating sections containing ACC (30 μm) were collected and subjected to immunohistochemistry using anti-LXRα, anti-LXRβ, anti-p65, anti-β-tubulin III, anti-Iba-1, anti-GFAP, anti-NeuN, anti-CAMK IIα, and anti-GAD67. Nuclei were counterstained with Hoechst 33258. Fluorescent signals were photographed and analyzed using confocal fluorescence microscopy (Olympus, Japan).

### Enzyme-linked immunosorbent assay

The levels of tumor necrosis factor-α (TNF-α), apolipoprotein E (ApoE) and ATP-binding cassette transporter (ABCA1) in blood and ACC were determined after the intraplantar injection of CFA with or without GW administration via enzyme-linked immunosorbent assay (ELISA) according to the manufacturer’s instructions.

### Whole-cell patch-clamp recording

Coronal slices (300 μm) containing ACC were prepared as described previously [17]. Slices were transferred to a recovery chamber with oxygenated (95% O_2_ and 5% CO_2_) artificial cerebrospinal fluid containing the following (in mM): 124 NaCl, 2.5 KCl, 2 CaCl_2_, 2 MgSO_4_, 25 NaHCO_3_, 1 NaH_2_PO_4_, and 10 glucose, for at least 1 h at room temperature. Excitatory postsynaptic currents (EPSCs) were recorded from layer II to III neurons of ACC with an Axopatch 200B amplifier (Axon Instruments, CA), and the stimulations were delivered in layer V of ACC. α-Amino-3-hydroxy-5-methyl-4-isoxazolepropionic acid (AMPA) receptor-mediated EPSCs were induced through repetitive stimulations at 0.02 Hz, and neurons were voltage clamped at − 70 mV. The recording pipettes (3–5 MΩ) were filled with solution containing the following (in mM): 145 K-gluconate, 5 NaCl, 1 MgCl_2_, 0.2 EGTA, 10 HEPES, 2 Mg-ATP, and 0.1 Na_3_-GTP, adjusted to pH 7.2 with KOH. For miniature EPSC (mEPSC) recording, TTX (0.5 μM) was added in the perfusion solution, and picrotoxin (100 μM) was always present to block GABA_A_ receptor-mediated inhibitory synaptic currents. Access resistance was 15–30 MΩ and monitored throughout the experiment. Data were discarded if access resistance changed more than 15% during an experiment.

### Co-immunoprecipitation

ACC was rapidly removed and washed in ice-cold PBS (0.1 mM) and lysed in NP-40 lysis buffer (Beyotime). The extract was pre-incubated with protein A/G PLUS-Agarose (Thermo Fisher) and normal rabbit IgG antibodies for 30 min at 4 °C. The mixture was then centrifuged at 2500 rpm for 5 min at 4 °C. For immunoprecipitation, equal amounts of protein were incubated with anti-p65, anti-LXRβ, or rabbit IgG at 4 °C for 2 h. Then, resuspended magnetic beads were added to each sample and incubated overnight at 4 °C. Magnetic beads were washed with lysis buffer three times and resuspended in SDS sample buffer. The immunoprecipitated protein complex were separated by SDS-PAGE and analyzed by immunoblotting using antibodies against p65 or LXRβ.

### RNA extraction and quantitative real-time PCR

Total RNA was isolated from neuron cultures using a standard method of phenol: chloroform extraction. cDNA was prepared using One Step SYBR® PrimeScript^TM^ RT-PCR Kit [26] according to the manufacturer’s instructions. Quantitative real-time PCR (qRT-PCR) was performed on the SYBR® Premix Ex Taq^TM^ in a Bio-Rad CFX96TM real-time PCR detection system (Bio-Rad) with the following thermocycling conditions: denaturation at 95 °C for 15 min, followed by 40 cycles of denaturation at 95 °C for 10 s and annealing at 55 °C for 30 s. Detection of the fluorescent product was carried out at the end of 95 °C extension incubation. PCR products were subjected to a melting curve analysis, and relative expression was calculated for each gene by the 2^-∆∆CT^ method [27]. All qRT-PCR reactions were performed in triplicate, and target gene expression was normalized to the levels of GAPDH mRNA. The primers used in this study were listed in Additional file 1: Table S1.

### Chromatin immunoprecipitation

Chromatin immunoprecipitation was performed using the EZ-Magna chip^TM^ A/G Kit (Millipore, 17-10086) [28]. Neurons grown on 60 mm^2^ culture dishes were cross-linked with 1% formaldehyde for 10 min at room temperature, followed by quenching the reaction with 0.125 M glycine. Samples were transferred to ice and washed with 1 ml PBS. The cells were incubated for 15 min on ice with 250 ml lysis buffer (including protease inhibitors). The lysate was sonicated to generate 200–1000 bp chromatin fragments. The supernatants after centrifugation were divided into three samples of 50 μl. Protein concentrations from different groups were measured by BCA assay and adjusted to the same protein level. Lysate was diluted 1:10 with dilution buffer (including protease inhibitors). Anti-AcH3 antibody (5 μl), anti-AcH4 antiserum (3 μl) or normal mouse IgG (1 μl), and protein A/G beads (20 μl) were mixed, and samples were rotated overnight at 4 °C. Beads were washed with washing buffer for four times. Chromatin complexes were eluted with 100 μl elution buffer (including Proteinase K), and the eluate was incubated at 62 °C for 2 h and 95 °C for 10 min. DNA was purified and analyzed by qRT-PCR using primers listed in Additional file 1: Table S1. Purified DNA sequencing was carried out by GeneCreate Biological Engineering Co., Ltd [29] and analyzed using Chromas 2.

### Data analysis

Results were analyzed by SPSS 19.0 and expressed as mean ± SEM. Differences between experimental groups were assessed by two-sample (unpaired Student’s) two-tailed *t* test assuming equal variance when comparing means between two groups; one-way ANOVA with least significant difference [5] test was used when comparing means between three or more groups; one-way ANOVA with Dennett’s T3 test was used when data were not passed the homogeneity test. Data of multiple groups were analyzed by two-way ANOVA followed by post hoc Tukey tests. In all cases, *p* < 0.05 was considered statistically significant.

## Results

### Dysfunction of LXRβ in ACC was required for developmental chronic pain mouse model

**Fig. 1:**
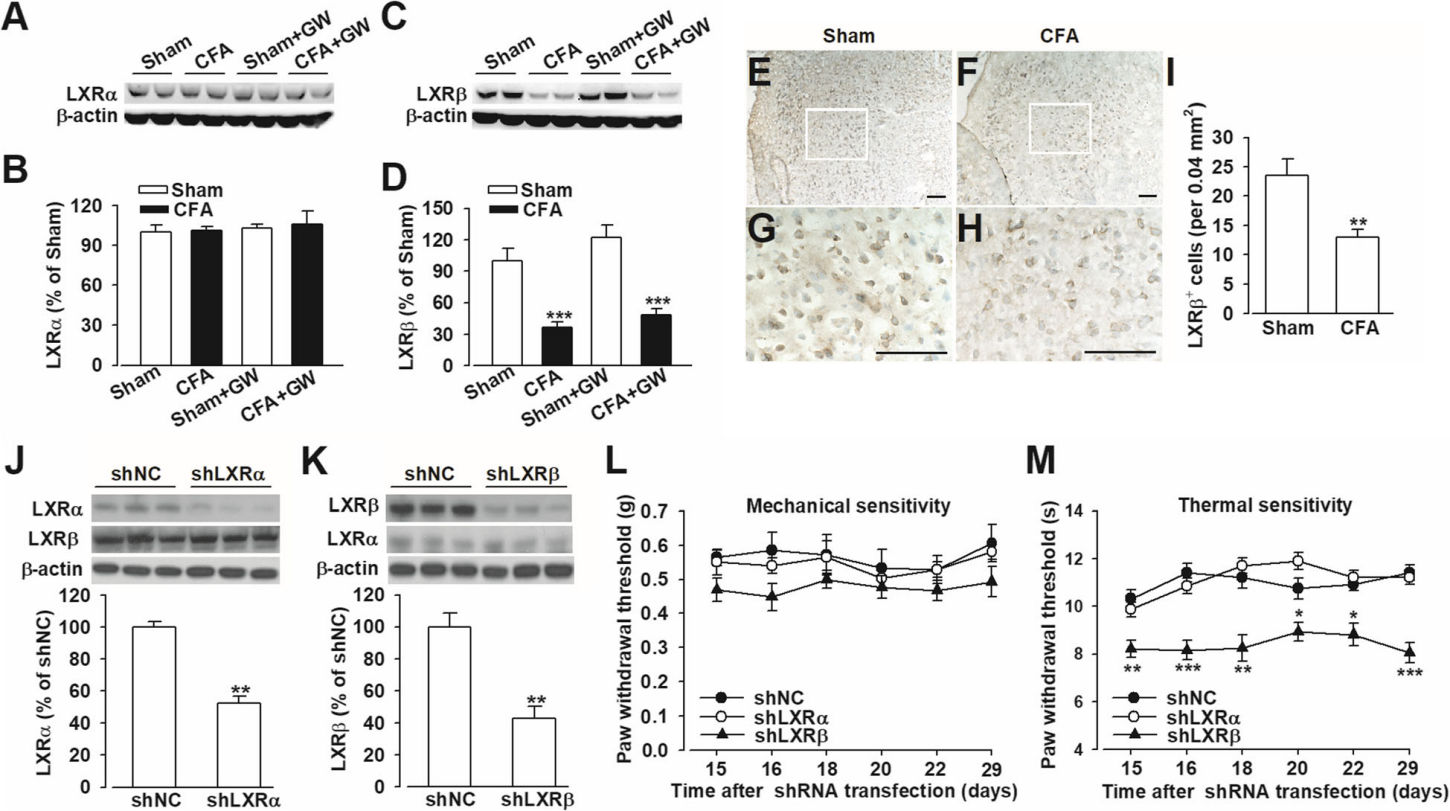
LXRβ deficit was associated with pain sensation in mice. Western blot analysis showed no changes of LXRα (**a, b)** and reduced LXRβ (**c, d**) in ACC after CFA injection. Representative examples of diaminobenzidine staining showed the expression of LXRβ immunoreactivity in ACC sections belonging to Sham (**e**) and CFA (**f**) mice. **g**, **h** Amplified views of the boxed areas in **e** and **f**. **i** Quantitative analysis of ACC sections showed the decreased LXRβ-positive cells in ACC from CFA-injected mice. Scale bars: **e** and **f**, 50 μm; **g** and **h**, 100 μm. **j**, **k** LXRα and LXRβ expression reduced after shRNA infection in ACC. **l** Mice with LXRβ knockdown did not show mechanical allodynia; however, **m** mice with LXRβ knockdown exhibited thermal hyperalgesia. Error bars represent SEM. *n* = 6 in **b** and **d**. *n* = 5 in **e**. *n* = 8 for shNC and shLXRα. *n* = 7 for shLXRβ group in **f***.* Error bars represent SEM. ^*^p < 0.05, ^**^p < 0.01, ^***^p < 0.001 *vs*. shNC-injected group

LXRs have been implicated in regulating cellular immunity and neuroinflammation, which may create a favorable microenvironment for anti-inflammation in the CNS. However, the role of LXRs in CIP is rarely reported. Immunofluorescent staining showed that LXRβ was widely expressed in ACC, whereas the expression of LXRα was very limited in ACC (Additional file 2: Figure S1). To determine whether LXRs signaling is required for chronic pain, complete Freund’s adjuvant (CFA) were injected into the hindpaws of mice to induce inflammatory pain. LXRα had low basal expression in ACC of naive mice and was not altered after CFA injection (*p* > 0.05; Fig. 1a, b), whereas LXRβ was relatively strongly expressed in ACC from healthy mice and substantially decreased in the CFA-treated mice (*p* < 0.001; Fig. 1c–i). And the reduction of LXRβ expression in ACC induced by CFA insult lasted to day 14 compared with the Sham group (*p* < 0.05, CFA vs. Sham; Additional file 3: Figure S2). These data suggested that LXRβ deficit in ACC may play an important role in the occurrence and maintenance of chronic pain. To further determine the roles of different LXR subtypes in the development of CIP, the expression levels of LXRα or LXRβ was knocked down via lentiviral shLXRα or shLXRβ transfection in the bilateral ACC, respectively. shLXR infection resulted in a reduction of LXRα protein to 52.1% ± 4.5% and LXRβ to 42.8% ± 7.5% of shNC (*p* < 0.01, *vs.* shNC; Fig. 1j, k). Both shLXRα and shNC groups presented no differences in response threshold to mechanical and thermal stimuli (*p* > 0.05, vs. shNC; Fig. 1l, m). However, shLXRβ mice exhibited hyperalgesia (*p* < 0.05, vs. shNC; Fig. 1m) but not allodynia (*p* > 0.05, vs. shNC; Fig. 1l). At the same time, no significant locomotor changes were observed in each group evaluated by open-field test and rotarod test after shRNA infection in ACC (*p* > 0.05, vs. shNC; Additional file 4: Figure S3). Collectively, LXRβ was involved in modulating the basal state of thermal sensitivity instead of mechanical nociception.

### LXRα/β dual agonist GW3965 inhibited the development of mechanical allodynia and thermal hyperalgesia after CFA injection in mice

**Fig. 2:**
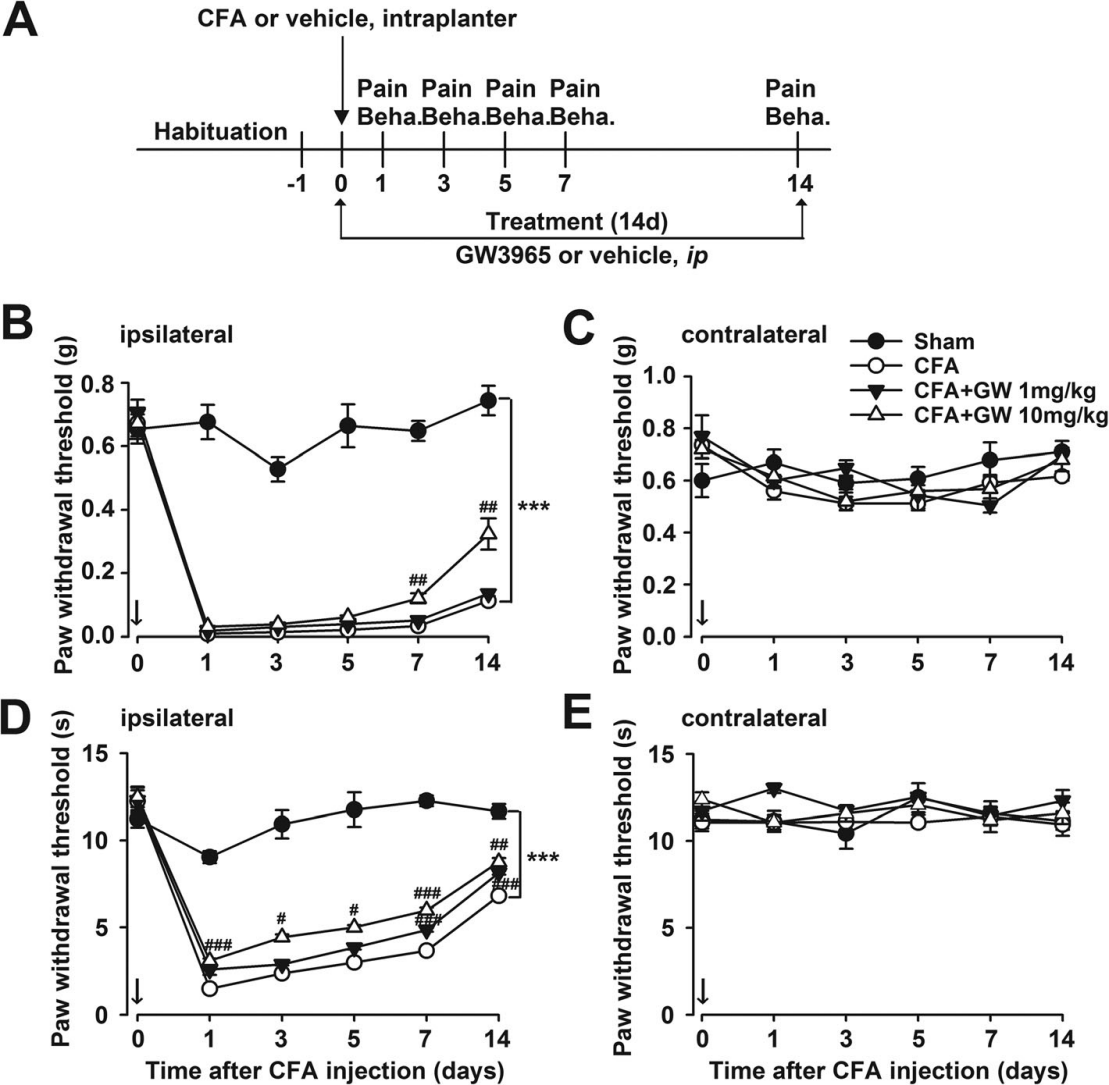
LXRβ activation by GW3965 reversed CFA-elicited persistent mechanical allodynia and thermal hyperalgesia. **a** Schematic illustration of the experiment procedure. **b**, **c** GW3965 (GW, 10 mg/kg) provided relief of mechanical allodynia on the ipsilateral side and no effect on the contralateral side in CFA-treated mice. **d**, **e** GW relieved thermal hyperalgesia on the ipsilateral side and had no effect on the contralateral side in CFA-treated mice. Error bars represent SEM. *n* = 5, ^*^p < 0.05, ^**^p < 0.01, ^***^p < 0.001 vs. Sham; ^#^*p* < 0.05, ^##^*p* < 0.01, ^###^*p* < 0.001 vs. CFA-injected group

The analgesic effects of LXR activation by GW3965 (GW) [11], an LXRα/β dual agonist, were tested according to Fig. 2a. CFA injection induced sensory hypersensitivity which was manifested as mechanical allodynia and thermal hyperalgesia (*p* < 0.001, CFA vs. Sham; Fig. 2b, d). GW administration (10 mg/kg, i.p) once per day immediately after CFA injection gradually relieved mechanical allodynia (*p* < 0.01, CFA + GW vs. CFA; Fig. 2b) and thermal hyperalgesia (*p* < 0.05, CFA + GW vs. CFA; Fig. 2d). However, GW showed a weak analgesic effect at lower dose of 1 mg/kg compared with that of 10 mg/kg. As a control, no pain behavior was observed in the contralateral hindpaw (*p* > 0.05; Fig. 2c, e). These results indicated that LXR activation by GW prevented the development of chronic pain.

### Isoform LXRβ mediated the analgesic effect of GW3965

**Fig. 3:**
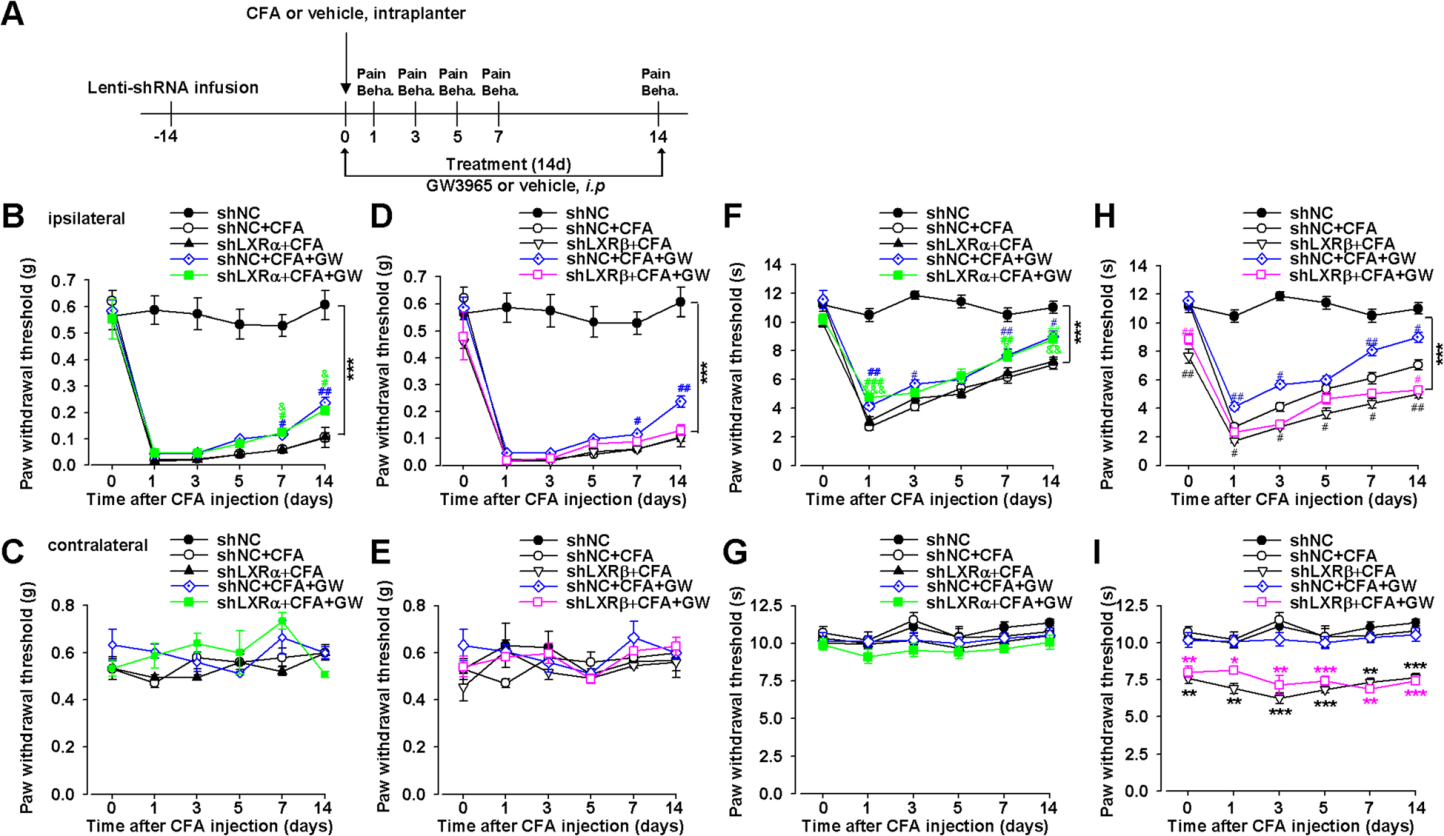
Knockdown of LXRβ expression in ACC by shLXRβ blocked GW3965-mediated analgesic effects in CFA-injected mice. **a** The schematic showed the experimental design for virus injection, drug treatment, and behavior tests. **b**, **c** LXRα knockdown by shLXRα did not affect GW-mediated analgesia of mechanical allodynia induced by CFA. **d**, **e** shLXRβ abolished GW-mediated analgesia of mechanical allodynia induced by CFA. **f**, **g** shLXRα did not affect GW-mediated analgesia of thermal hyperalgesia induced by CFA. **h**, **i** LXRβ knockdown by shLXRβ abolished GW-mediated analgesia of thermal hyperalgesia induced by CFA. Error bars represent SEM. *n* = 6, ^*^p < 0.05, ^**^p < 0.01, ^***^p < 0.001 vs. shNC-injected group; ^#^*p* < 0.05, ^##^*p* < 0.01 *vs*. shNC + CFA group; ^&^*p* < 0.05, ^&&^*p* < 0.01, ^&&&^*p* < 0.001 vs. shLXRα + CFA group

Given the lack of selective agonists for LXRα and LXRβ isoforms, we applied specific shRNA-mediated knockdown of LXRs in ACC to test the roles of LXRα and LXRβ in the analgesic effects of GW (Fig. 3a). Knockdown of LXRβ expression by lentiviral microinjection into bilateral ACC completely eliminated GW-mediated analgesic effects (*p* > 0.05, shLXRβ + CFA + GW vs. shNC + CFA group; Fig. 3d, h), whereas lentiviral shLXRα did not affect GW-mediated analgesic effects in CIP mice (*p* < 0.05, shLXRα + CFA + GW vs. shLXRα + CFA; Fig. 3b, f), suggesting that LXRβ isoform mediated the analgesic effect of GW. Consistent with preceding experiments, lentiviral shLXRβ but not shLXRα decreased withdrawal latency of thermal pain in the contralateral hindpaw of mice (*p* < 0.05, shLXRβ vs. shNC; *p* > 0.05, shLXRα vs. shNC; Fig. 3g, i). Moreover, lentiviral shLXRβ enhanced CFA-induced hyperalgesia (*p* < 0.05, shLXRβ + CFA vs. shNC + CFA; Fig. 3h), confirming LXRβ isoform was involved in CIP development.

### Reduced nuclear factor-κB transcriptional activity in CFA-induced inflammatory chronic pain after GW3965 treatment

**Fig. 4:**
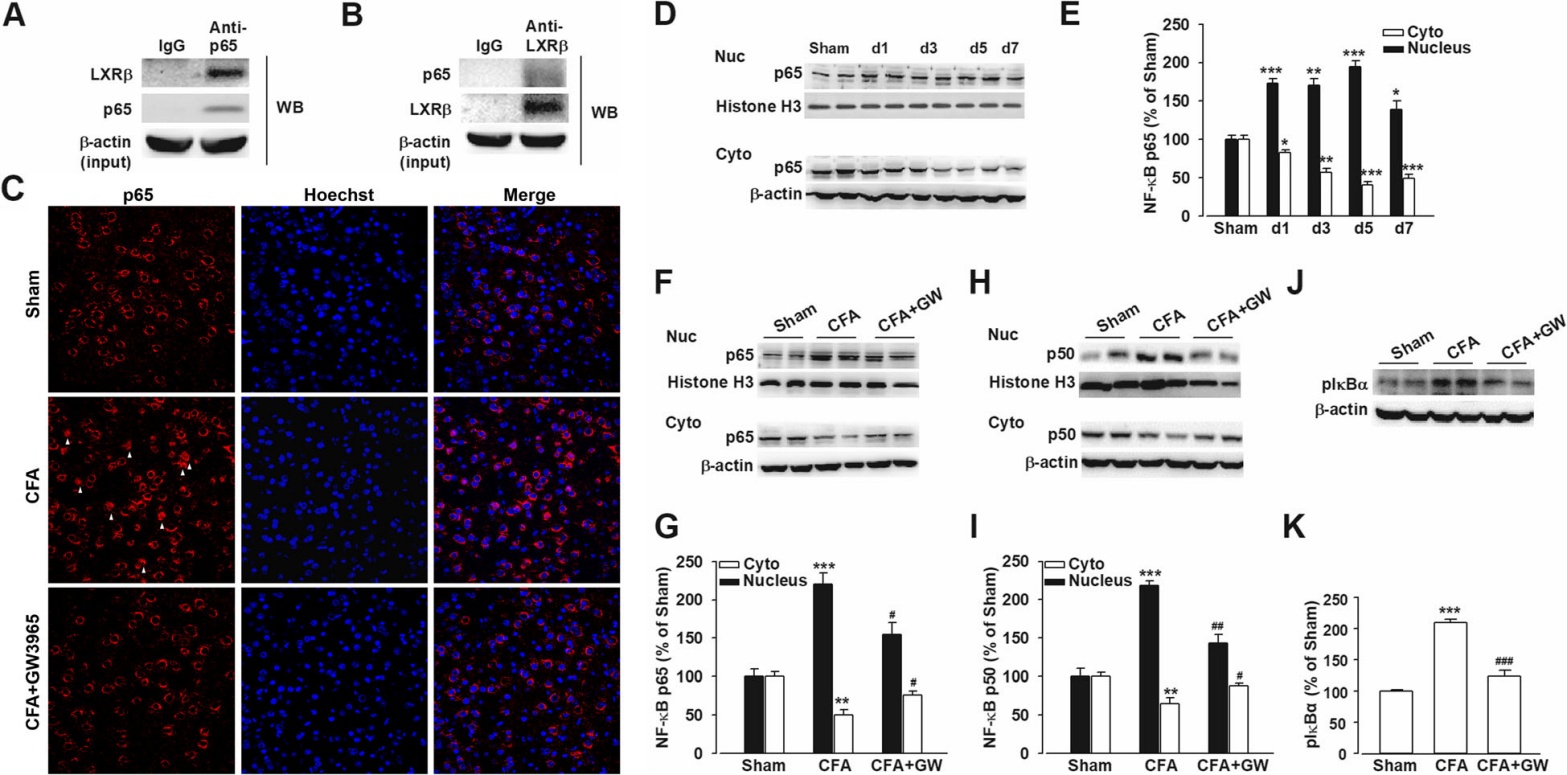
GW3965 inhibited the increased nuclear translocation of NF-κB and MAPK phosphorylation in mice ACC after CFA injection. **a**, **b** Co-IP revealed the interaction of endogenous LXRβ with NF-κB p65 in mouse ACC. **c** Representative images of nuclear translocation of NF-κB p65 in ACC from Sham, and mice after CFA injection with or without GW3965 administration (GW, 10 mg/kg) were stained for p65 (red) and Hoechst (blue). **d**, **e** The time course of p65 nuclear translocation in ACC after CFA insult. GW treatment (10 mg/kg) reversed the enhanced nuclear translocation of p65 (**f**, **g**), p50 (**h**, **i**), and p-IκBα expression (**j**, **k**) after CFA injection. *n* = 5, ^*^p < 0.05, ^**^p < 0.01, ^***^p < 0.001 vs. Sham; ^#^*p* < 0.05, ^###^*p* < 0.001 vs. CFA-injected group

Previous work reported that the anti-inflammatory effect of LXRs activation was mediated by NF-κB inhibition [30–32]. Co-IP assay validated that LXRβ physically interacted with NF-κB p65 in the multiprotein complexes extracted from ACC (Fig. 4a, b). CFA injection induced an increase level of nuclear p65 and a decrease expression of cytoplasmic p65 correspondingly in ACC (Fig. 4d, e)*.* GW treatment obviously reversed the enhanced nuclear translocation of p65 and p50 (*p* < 0.05, CFA + GW vs. CFA; Fig. 4c, f–i), which compose the most common heterodimer of NF-κB. At the same time, GW administration corrected the elevated p-IκBα (phosphorylated NF-κB inhibitor alpha) (*p* < 0.001, CFA + GW vs. CFA; Fig. 4j, k), which is a major indicator of NF-κB activity after CFA injection [33]. Collectively, GW administration inhibited the NF-κB transcriptional activity and thus may suppress the inflammatory responses.

### GW3965 administration suppressed CFA-induced neuroinflammation

**Fig. 5:**
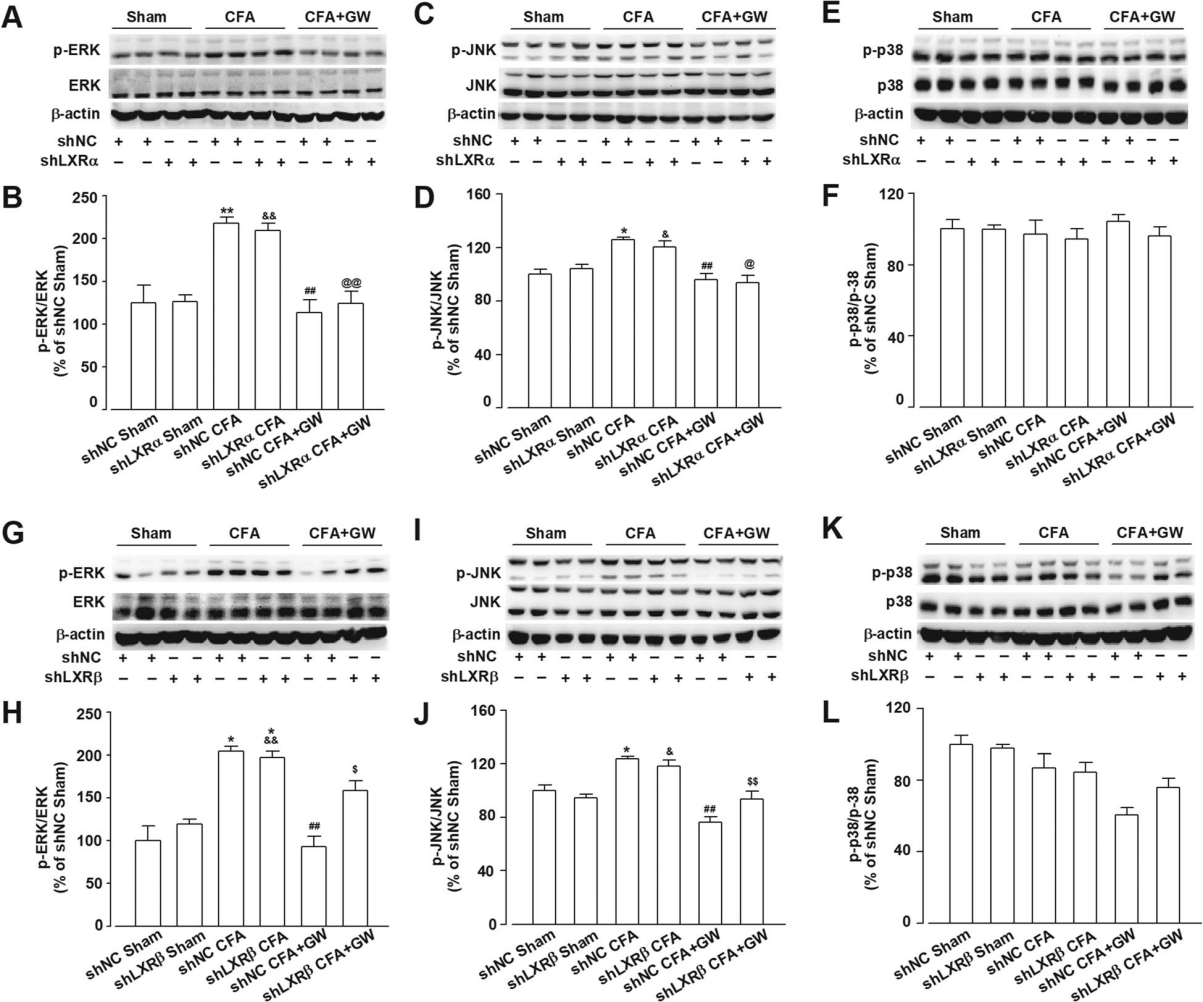
shLXRβ but not shLXRα abolished GW-mediated inhibition of phosphorylated MAPKs. Western blot analysis revealed shLXRα did not affect GW-mediated inhibition of elevated p-ERK (**a**, **b**) and p-JNK (**c**, **d**) in ACC induced by CFA and had no impact on p-p38 expression (**e**, **f**). However, shLXRβ abolished GW-mediated inhibition of p-ERK (**g**, **h**) and p-JNK (**i**, **j**) but did not affect p-p38 expression in CIP mice (**k**, **l**). Error bars represent SEM. *n* = 4, ^*^p < 0.05, **p < 0.01 vs. Sham or shNC + Sham group; ^#^*p* < 0.05 vs. shNC + CFA group; ^&^*p* < 0.05, ^&&^*p* < 0.01 vs. shLXRβ + Sham; ^@^*p* < 0.05, ^@@^*p* < 0.01 vs. shLXRα + CFA; ^$^*p* < 0.05, ^$$^*p* < 0.01 vs. shNC + CFA + GW

Accumulating evidence indicates that the phosphorylated form of mitogen-activated protein kinase (MAPK) is involved in the induction and maintenance of both acute and inflammatory pain [34, 35]. CFA injection induced a higher amount of p-ERK and p-JNK but not p-p38 expression in both shNC and shLXRs microinjection mice (*p* < 0.05, shNC + CFA vs. shNC + Sham; shLXRα + CFA vs. shLXRα + Sham; shLXRβ + CFA vs. shLXRβ + Sham; Fig. 5). GW administration could inhibit the activated kinases (*p* < 0.01, shNC + CFA + GW vs. shNC + CFA; Fig. 5a–d, g–j). Consistent with the behavior results, the downregulation of LXRβ by shLXRβ partially abolished the effects of GW on MAPK phosphorylation, including p-ERK and p-JNK (*p* > 0.05, shLXRβ + CFA + GW vs. shLXRβ + CFA; Fig. 5g–j). However, the downregulation of LXRα by shLXRα did not affect GW-mediated inhibition of MAPK phosphorylation (*p* < 0.05, shLXRα + CFA + GW vs. shLXRα + CFA; Fig. 5a–d). Thus, the reduction of activated MAPKs by GW via LXRβ activation alleviated pain behaviors in CFA-insulted mice.

Our previous study indicated that TNF-α in ACC was involved in CIP development [36]. As expected, GW treatment reversed the increase of TNF-α in ACC after CFA exposure (*p* < 0.05, CFA + GW d1, d3 vs. CFA d1, d3 in serum; *p* < 0.05, CFA + GW d1, d3, d5 vs. CFA d1, d3, d5 in ACC; Additional file 5: Figure S4a, b). The expression of ABCA1 and ApoE, which are LXR target genes, was determined to measure LXRs activation upon GW stimulation. ApoE expression was increased promptly from d1 to d7 both in serum and ACC after GW treatment (*p* < 0.05, CFA + GW d1, 3, 5, 7 vs. CFA d1, 3, 5, 7 in serum; *p* < 0.05, CFA + GW d3, d5 vs. CFA d3, d5 in ACC; *p* < 0.01, CFA + GW d1, d7 vs. CFA d1, d7; Additional file 5: Figure S4c, d); ABCA1 expression in ACC increased dramatically at d1 after GW administration, while the increase of ABCA1 in serum showed significant at d5 and d7 after GW administration (*p* < 0.05, CFA + GW d7 vs. CFA d7 in serum, *p* < 0.01, CFA + GW d5 vs. CFA d5 in serum; *p* < 0.001, CFA + GW d1, d5, d7 vs. CFA d1, d5, d7 in ACC; Additional file 5: Figure S4e, f). Thereby, the data indicated that LXRs were activated in a ligand-dependent manner. Collectively, these data suggested that LXR activation suppressed CFA-induced inflammation.

### GW3965 administration suppressed the enhanced synaptic transmission in the ACC from CFA-injected mice

**Fig. 6:**
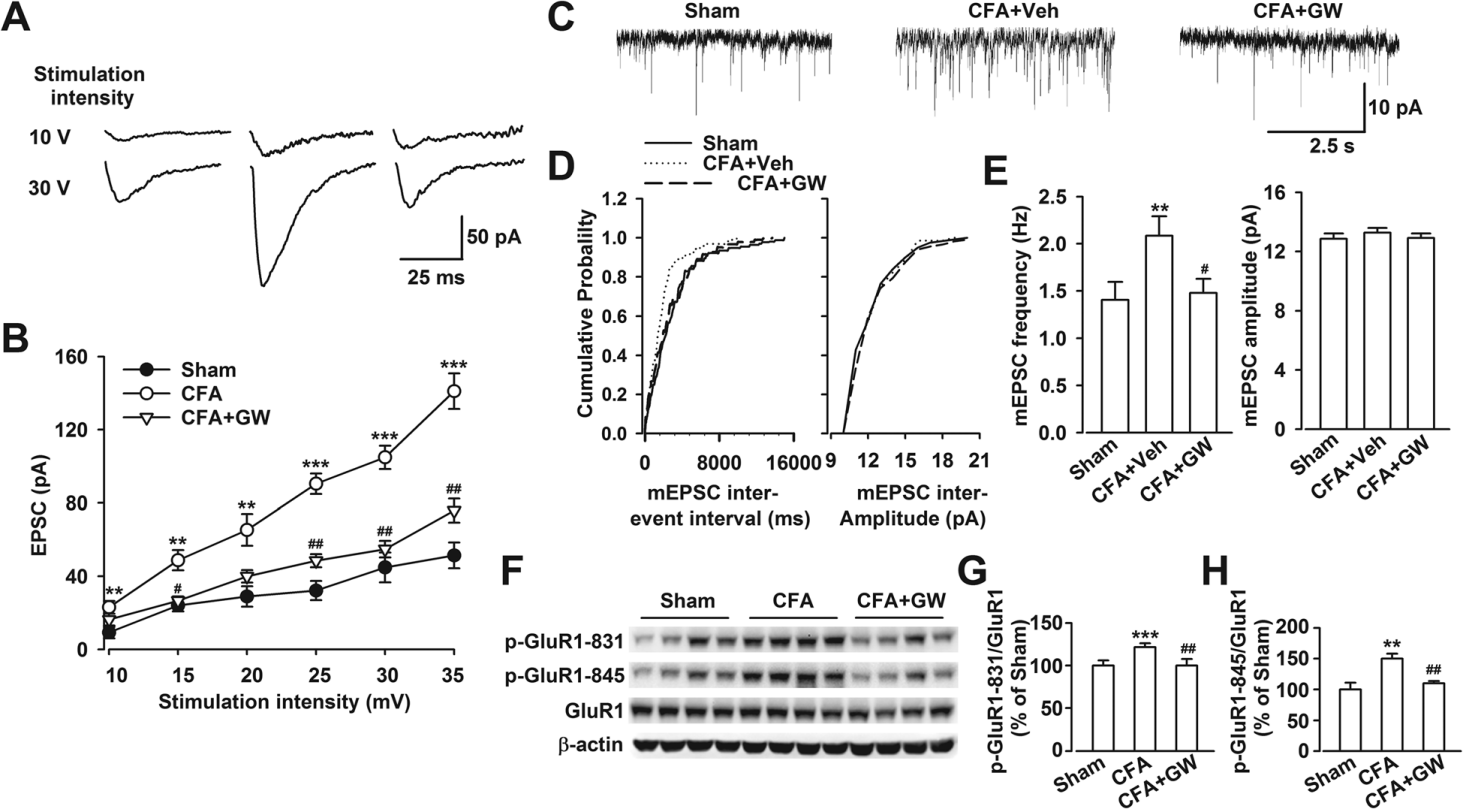
GW3965 infusion abolished the enhanced excitatory transmission in CFA-injected mice. **a** Representative traces showed averages of EPSCs at 10 and 30 V stimulation intensity in mice ACC. **b** Plot of input-output curves showed GW3965 (GW, 10 μM) perfusion partially abolished the enhancement of synaptic transmission in ACC of CFA-injected mice. *n* = 6 neurons/3 mice. **c** Representative mEPSCs recorded in pyramidal neurons of ACC at a holding potential of − 70 mV. **d** Cumulative frequency (left) and amplitude (right) histogram of the mEPSCs from the cells in each group. **e** Quantitative analysis of neurons in ACC from each group showed GW incubation blocked the enhanced mEPSC frequency (left) induced by CFA, while GW had no effect on mEPSC amplitude (right). *n* = 7 neurons/3 mice in control, *n =* 9 neurons/3 mice in CFA + vehicle, and *n =* 9 neurons/3 mice in CFA + GW mice. **f** GW treatment (10 mg/kg) reversed enhanced phosphorylation of GluR1 at **g** Ser831 (p-GluR1-S831) and **h** Ser845 (pGluR1-S845) induced by CFA injection. Error bars represent SEM. *n =* 6, ^**^p < 0.01, ^***^p < 0.001 vs. Sham group, ^#^*p* < 0.05, ^##^*p* < 0.01 vs. CFA-injected group

Previous studies evidenced that persistent inflammatory pain caused by CFA was due to an enhancement in excitatory synaptic transmission in ACC [17]. The expression pattern showed that LXRβ colocalized mainly in neuron (β-tubulin III positive) and less in astrocyte (GFAP positive) and microglia (Iba-1 positive) in ACC (Additional file 6: Figure S5a–c). LXRβ-positive neurons were mainly distributed in glutamatergic neurons (CAMK IIα positive) and moderately in GABAergic neurons (GAD67 positive) as shown in Additional file 6: Figure S5d, e. To demonstrate the possible mechanisms of LXRβ in CIP, excitatory postsynaptic currents (EPSCs) were recorded from layer II to III neurons of ACC. The slope of the input-output curve was significantly potentiated in slices from CFA-injected mice compared with Sham, which was obviously reduced by GW incubation (*p* < 0.01, CFA vs. Sham; *p* < 0.05, CFA + GW vs. CFA; Fig. 6a, b), suggesting that LXRs activation reversed the increased synaptic efficacy in ACC neurons from CFA-injected mice. To further explore the role of GW in synaptic transmission after CFA injection, AMPA receptor-mediated mEPSCs, reflecting the release of single quanta of neurotransmitter, were measured. As expected, GW incubation could reverse the strengthened mEPSC frequency induced by CFA (*p* < 0.05, CFA + GW vs. CFA + Veh; Fig. 6c–e). These findings provided evidence that GW inhibited the excitatory glutamatergic neurotransmission which was involved in pain development through LXRs activation. It is well evidenced that GluR1-containing AMPA receptors play crucial roles in pain and emotional disorders, especially the phosphorylation of GluR1 at Ser831/Ser845. We further found that GW administration reversed the hyperphosphorylation of GluR1 at Ser831 and Ser845 (*p* < 0.01, CFA + GW vs. CFA; Fig. 6f–h) in mice ACC upon CFA injury.

### Reduction of LXRβ expression in CFA mice was associated with enhanced activity of HDAC5

**Fig. 7:**
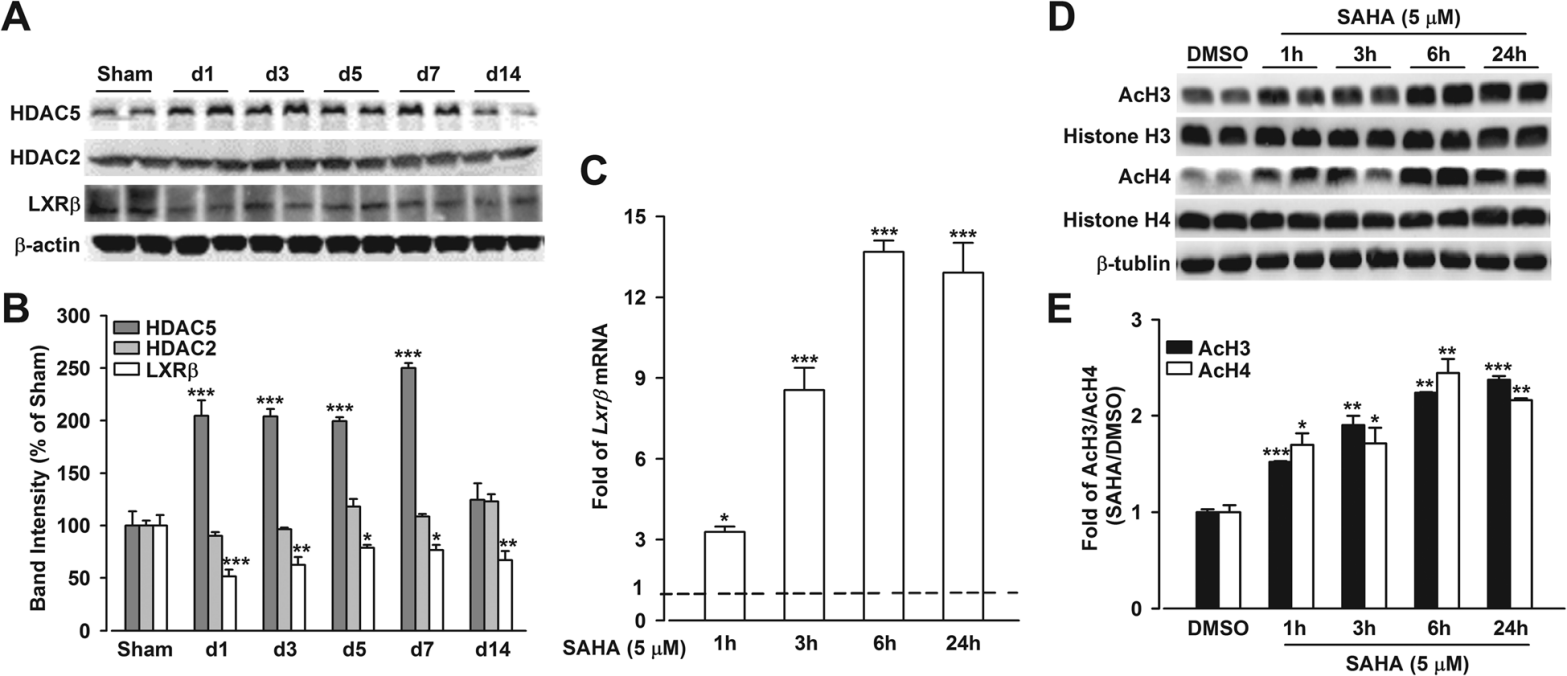
Enhanced acetylated histone 3 (AcH3) and histone 4 (AcH4) were responsible for LXRβ induction by inhibiting HDAC5 activity. **a**, **b** Western blot analysis revealed that upregulated expression of HDAC5 but not HDAC2 was accompanied by LXRβ reduction in ACC after CFA insult. *n* = 4, ^*^p < 0.05, ^**^p < 0.01, ^***^p < 0.001 vs. Sham group. **d, e** SAHA incubation led to the enhanced expression of AcH3 and AcH4 in cultured neurons. **c** SAHA (5 μM) incubation induced *Lxrβ* mRNA expression in cultured neurons, *n* = 5. qRT-PCR data were normalized to *Gapdh* expression and were expressed as induction fold relative to DMSO-treated control (dotted line), *n* = 10. Error bars represent SEM. ^*^p < 0.05, ^**^p < 0.01, ^***^p < 0.001 vs. DMSO-treated control

Evidence deciphered that epigenetic regulation plays a critical role in CIP development, and we revealed that the dysfunction of LXRβ induced by CFA insult led to mice analgesia. Thus, we further explored the epigenetic mechanisms on LXRβ regulation. Western blot analysis showed HDAC5 expression (*p* < 0.001, CFA *vs.* Sham; Fig. 7a, b) but not HDAC2 expression elevated upon CFA injury (*p* > 0.05, CFA vs. Sham; Fig. 7a, b); HDAC5 expression was negatively correlated with that of LXRβ in ACC (*p* < 0.05, CFA *vs.* Sham; Fig. 7a, b), suggesting that epigenetics might intervene in the expression of *Lxrβ* gene, accompanied by pain sensation induced by CFA. To determine whether HDAC inhibited *Lxrβ* expression, an in vitro culture system of neurons was applied. Incubation of cultured neurons with SAHA (5 μM), a class I/IIb HDAC inhibitor, led to an induction of *Lxrβ* mRNA expression (*p* < 0.05, SAHA vs. DMSO; Fig. 7c), suggesting SAHA restored *Lxrβ* gene expression by inhibiting HDAC activity. Meanwhile, SAHA induced global histone acetylation, including AcH3 and AcH4 levels in cultured neurons (*p* < 0.05, SAHA vs. DMSO; Fig. 7d, e), indicating that enhanced AcH3 and AcH4 were responsible for *Lxrβ* induction. These data indicated that *Lxrβ* expression was regulated by HDAC5.

**Fig. 8:**
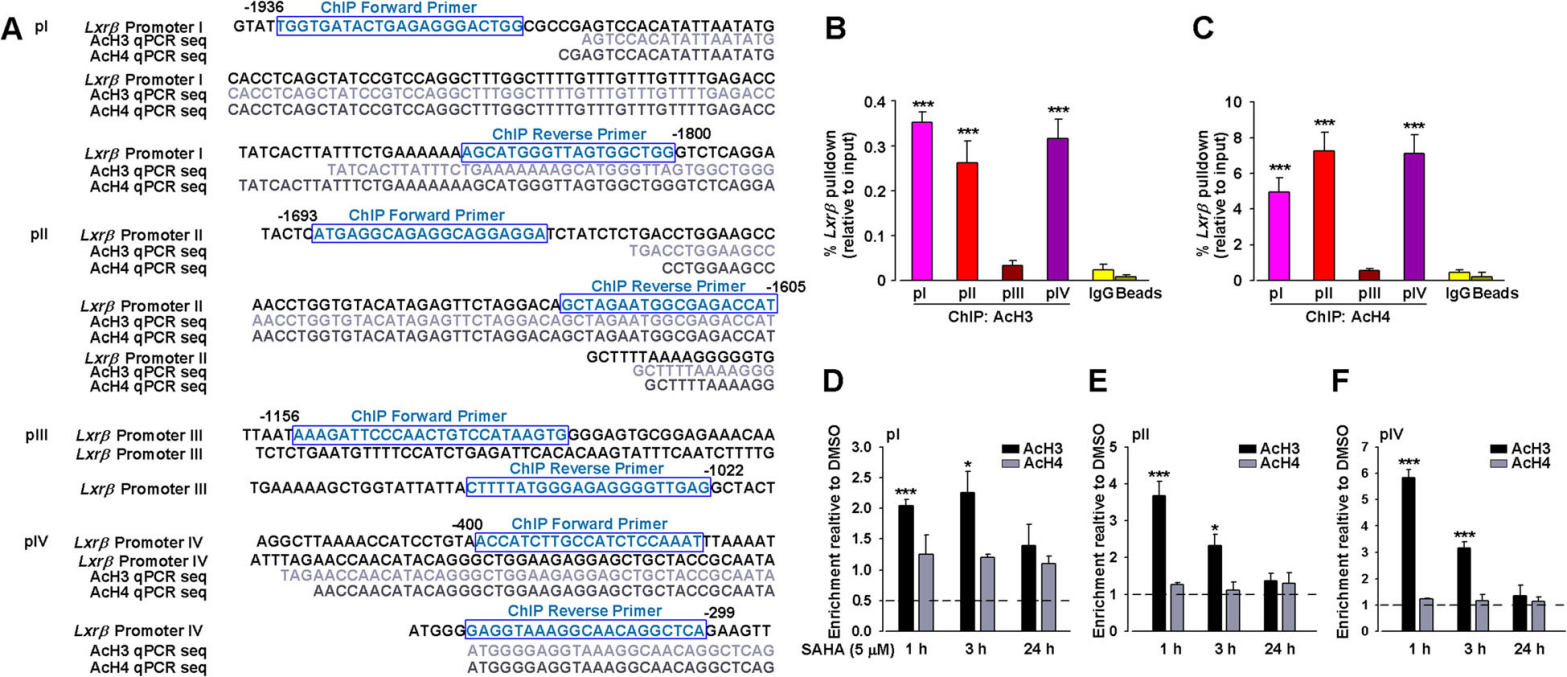
Epigenetic regulation of *Lxrβ* by class I/IIb HDAC inhibitor SAHA in the cortical cultured neurons. Signal-to-noise determination of acetylated histone binding sites on *Lxrβ* promoter. **a** Sequencing of ChIP-qPCR material obtained from cultured neurons showed acetylated histones (AcH3 or AcH4) bound to *Lxrβ* promoter I, II, and IV. *Lxrβ* promoter enrichment (shown as % of input) displayed alongside IgG control ChIPs for **b** AcH3 and **c** AcH4. *n* = 6, ^***^p < 0.001 vs IgG negative control. The boxes indicated the forward or reverse promoter sites of ChIP. SAHA treatment increased the binding of AcH3 but not AcH4 on *Lxrβ* promoter I (**d**), II (**e**), and IV (**f**). Results were normalized with input levels and were expressed as enrichment relative to DMSO-treated control (dotted lines). Error bars represent SEM. *n* = 5, ^*^p < 0.05, ^***^p < 0.001 vs. DMSO-treated control

Finally, to demonstrate the impacts of histone modifications on gene expression, a promoter analysis of *Lxrβ* was performed to identify the potential regulatory regions where acetylated histones might bind. The total length of 2000 bp upstream of the *Lxrβ* transcription start site was analyzed, and four pairs of highly specific primers for *Lxrβ* were designed. ChIP analysis was carried out to test the enrichment of acetylated histones at *Lxrβ* promoter region in cortical neurons with or without SAHA treatment, validating by comparison to IgG control ChIP and promoter amplicon sequencing (Fig. 8a). Analysis revealed that AcH3 and AcH4 were bound to the regions of about 1900 bp, 1600 bp, and 350 bp (*p* < 0.001, pI, II, IV vs. IgG; Fig. 8b, c) but not 1100 bp (*p* > 0.05, pIII vs. IgG; Fig. 8b, c) upstream of the transcription start site of *Lxrβ* in cultured neurons.

Next, we asked if the dynamics of *Lxrβ* mRNA induction by HDAC inhibition correlated with histone acetylation at respective promoters. We analyzed the expression levels of AcH3 and AcH4 at these promoters after SAHA treatment. Surprisingly, the levels of AcH3 but not AcH4 at *Lxrβ* promoter I, II, and IV were robustly induced by SAHA treatment for 1 h, followed by a gradual decrease in 24 h after SAHA treatment (*p* < 0.001, SAHA 1 h, 3 h vs. DMSO; *p* > 0.05, SAHA 24 h vs. DMSO for AcH3; *p* > 0.05, SAHA vs. DMSO for AcH4; Fig. 8d–f). This was in sharp contrast with the relative induction of *Lxrβ* mRNA by SAHA treatment. Taken together, the epigenetic suppression of LXRβ in ACC by HDAC drove CFA-induced pain.

## Discussion

**Fig. 9:**
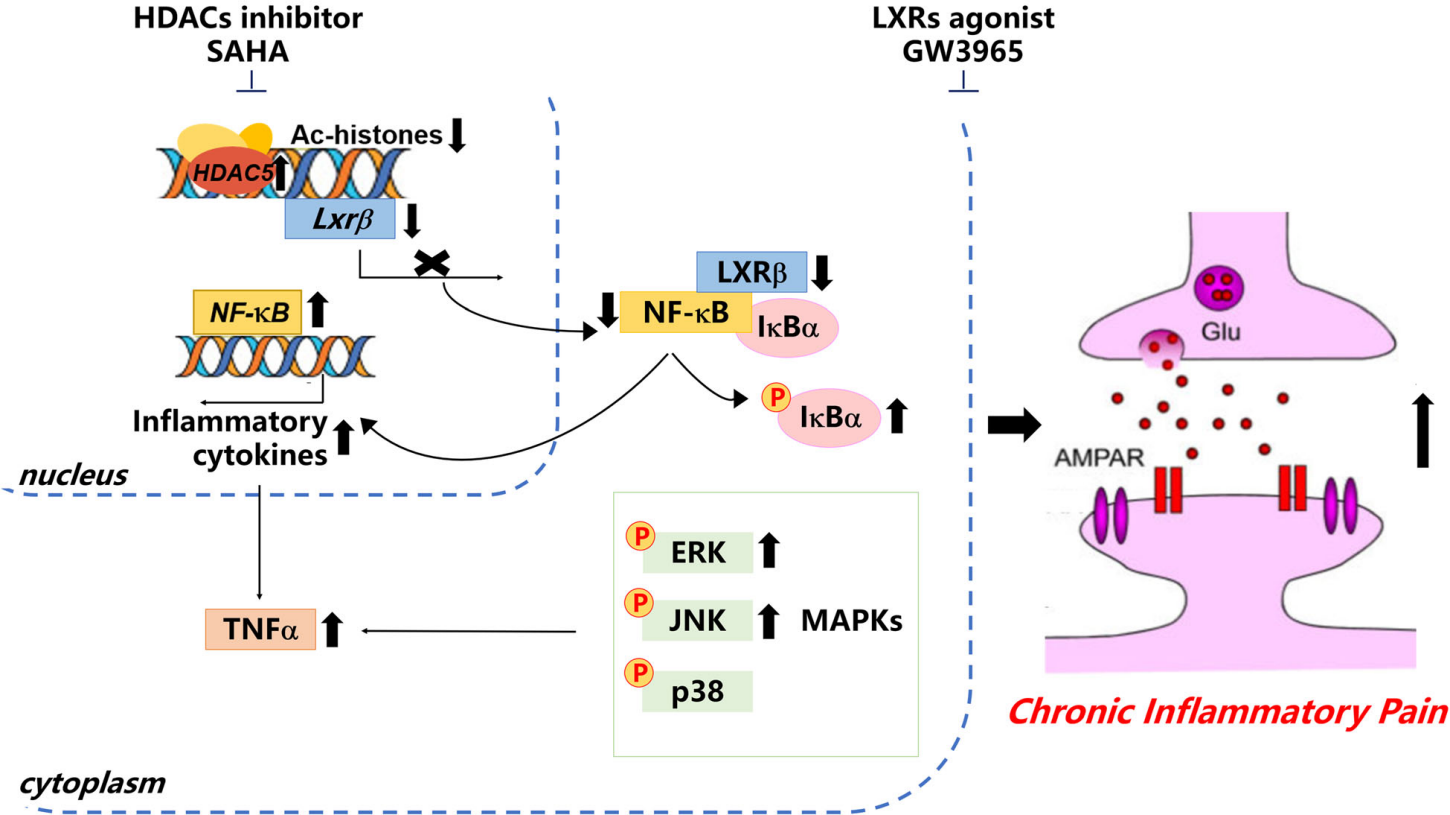
Conceptual summary. HDAC5 was activated upon CFA insult, resulting in reduced expression of AcH3 and AcH4 on *Lxrβ* promoter, which contributed to the reduction of LXRβ expression. SAHA treatment sequestered HDACs that promoted AcH3 expression on *Lxrβ* promoter and LXRβ expression. LXRβ activation by GW3965 was able to reverse the nuclear translocation of p65 and p50, reduce the phosphorylation of mitogen-activated protein kinases (MAPKs) signaling pathways, regulate the TNF-α expression, and modulate the enhanced excitatory synaptic transmission in CFA-induced chronic inflammatory pain

In the current study, we have shown that LXRβ levels in ACC were reduced in mice with CFA-induced chronic pain and LXRβ knockdown in ACC led to pain sensation behaviors. LXRβ activation exerted analgesic effects through anti-inflammation and rebalancing the neurotransmission in CFA-induced pain model. ChIP analysis revealed that HDAC5 triggered histone deacetylation on the promoter region of *Lxrβ*, resulting in the downregulation of *Lxrβ* transcription. Thus, our results suggest LXRβ activation may represent a potential novel target for the treatment of chronic pain and also provide a novel insight into the epigenetic mechanism underlying mechanisms of chronic pain (Fig. 9).

LXRβ mutation was involved in acute and inflammatory pain [37]. It has been demonstrated that activation of LXRs by GW, a synthetic full agonist for both LXRα and LXRβ isoforms, which can readily cross the blood-brain barrier to exert its specific actions in brain, exerted an antinociceptive effect in rat joint pain and diabetes-caused thermal hyperalgesia [38, 39], suggesting that the dysfunction of LXRs contributes to pain pathogenesis. We also found that CFA induced the reduction of LXRβ levels in mice ACC along with hyperalgesia (Fig. 1). And LXR agonist, GW administration did not change the expression of LXRs, indicating that the GW-mediated inhibition of mechanical allodynia and thermal hyperalgesia was through activating LXRβ but not the expression of LXRβ. Moreover, the knockdown of LXRβ levels in ACC by shRNA led to thermal hyperalgesia, which manifested as shorter latencies in a plantar analgesia device, further implying that the downregulation of LXRβ in ACC contributed to the etiology of chronic pain. Moreover, we first demonstrated GW administration reversed CFA-induced pain behaviors (Fig. 2), confirming the analgesic effects of GW. Given the lack of LXRβ isoform-specific agonist and antagonist, we used shRNA-mediated knockdown of LXRβ isoform to further validate the roles of LXRβ in the analgesic effects of GW. Microinjections of the shLXRβ into ACC bilaterally before GW treatment completely abrogated the analgesic effects of GW (Fig. 3), thereby indicating that LXRβ isoform mediated the analgesic effects of GW. Furthermore, LXRβ was also expressed in the spinal cord, and male LXRβ^-/-^ mice suffered from adult-onset motor neuron degeneration, whereas LXRβ activation by T0901317 and GW protected the spinal cord from injury. Our results showed downregulated LXRβ in bilateral ACC, and GW-mediated analgesic effect was almost blocked, indicating the important role of LXRβ of ACC in pain modulation. We cannot exclude the effects of GW mediated by activating endogenous LXRs in the spinal cord by* i.p* administration. Therefore, the different routes of GW administration, such as intrathecal or intraventricular injection, could be further conducted and explored to confirm the activated effects of GW on ACC LXRβ in pain regulation.

Evidence indicated the epigenetic mechanisms controlling central sensitization for persistent pain, especially histone acetylation/deacetylation. Histone acetylation is often associated with many transcription factors, archiving a given pattern of gene expression, whereas deacetylation is thought to suppress gene expression. HDAC inhibitors (HDACi) or increase of histone acetylation is able to attenuate persistent pain-associated inflammation [18, 40], supporting the analgesic effect of histone acetylation which was involved in the development of chronic pain. HDAC5 is a crucial member of class I HDACs and a promising candidate in pain modulation, and we observed the downregulation of LXRβ was associated with HDAC5 induction in ACC from CFA-insulted mice (Fig. 7a, b). Inhibition of HDAC5 by SAHA in cultured neurons led to the induction of AcH3 and *Lxrβ* (Fig. 7c–e), and ChIP data showed a significant binding of histone acetylation to the promoters of *Lxrβ* (Fig. 8), indicating that LXRβ was a downstream target of HDAC5 in ACC of CIP mice. This data suggested that HDAC5 induced the deacetylation of *Lxrβ* gene with high selectivity, providing direct evidence that suppression of LXRβ in CIP was potentially regulated by histone acetylation/deacetylation. In this study, the HDAC5 participation in the suppression of LXRβ in CIP was specifically confirmed for the first time.

Central sensitization is triggered by NF-κB-related proinflammatory mediators, including the cytokines TNF-α and IL-1β, which can act directly on their receptors or targets to reduce pain thresholds, leading to inflammatory pain and hyperalgesia [36, 41]. We found that activation of LXRβ by GW decreased the CFA-induced production of cytokines such as TNF-α (Additional file 5: Figure S4) and NF-κB (Fig. 4) in ACC. Thus, we infer that an LXRβ-dependent mechanism regulating the synthesis or release of pro-inflammatory chemokines such as TNF-α through NF-κB pathway may mediate the inflammatory response to CFA. This might explain reduced pain response by GW treatment in CFA test.

MAPK family is phosphorylated upon various types of noxious stimuli [35, 42]. Accumulating evidence indicates that exaggerated inflammatory nociception has been associated with ERK and JNK activation in different pain-relevant tissues during acute and chronic experimental pain [43]. In the current study, we demonstrated that GW treatment completely abolished the induction of p-ERK and p-JNK upon CFA injury (Fig. 5), thereby implying that inhibition of inflammation may also be involved in the analgesic effects mediated by LXRβ activation.

Recent studies from animals and humans demonstrate the critical importance of ACC neurons in behavioral nociceptive responses to injury, whereas it governs or integrates pain perception or unpleasant moods in humans [44, 45]. Indeed, ACC is one of the key nodes in a complex network of cortical and subcortical structures that process and regulate pain behavior and aversion. Chronic pain increases the basal and noxious stimulus-induced firing rates in ACC; thus, an enhancement of neuron activities in ACC may play complementary roles in the chronic pain state [46, 47]. Inflammatory stimuli lead to the consequent modulation of synaptic transmission and neuronal excitability, which are involved in pain facilitation [48]. Our study is the first time to examine whether LXR activation reversed altered synaptic transmission in ACC of mice with CIP. Our previous study demonstrated an enhancement of synaptic transmission in ACC of animals suffering from CIP [17]. Our results demonstrated that GW abolished the increased excitatory neurotransmission in ACC induced by CFA (Fig. 6). Collectively, we provide evidence for the roles of LXR-mediated analgesic effects, which were likely to be achieved through both anti-inflammation and neurotransmitter rebalance.

Moreover, evidence links endoplasmic reticulum stress (ERS) to chronic pain [49–51], especially obesity-induced allodynia in mice, while the activation of LXRs alleviates the allodynia through delaying ERS [52]. We also found that GW administration reversed the activated ERS in CIP mice, measured by the enhanced expression of ERS markers, C/EBP homologous protein (CHOP), and activating transcription factor 4 (ATF4) in ACC (Additional file 7: Figure S6) by qPCR, indicating that LXR activation attenuated ERS in CIP.

Despite the potential analgesic effects, unfortunately, LXRα/β dual agonists, including GW, may elevate hepatic and serum triglyceride levels. Elevated plasma or hepatic triglyceride levels are unacceptable side effects for LXRα/β dual agonists due to the risks of cardiovascular diseases and hepatotoxicity. LXRα is the main isoform responsible for the deleterious triglyceride-raising effects of full LXR agonists. Therefore, developing selective agonists for LXRβ isoform to eliminate the adverse effect of increased triglyceride is still needed. Our findings support that LXRβ levels in ACC were negatively correlated with pain behaviors. The analgesic effects mediated by LXRβ activation were likely through anti-inflammation pathways. Thus, the reduced LXRβ level by epigenetic regulation may be implicated in the etiology of chronic pain, and LXRβ activation may represent a potential novel target for the treatment of chronic pain.

## Conclusion

In conclusion, our study reveals the analgesic effects of LXRβ activation are associated with the regulation of neuroinflammation and central sensitization in CFA-induced inflammatory pain. Our research provides, for the first time, a novel epigenetic mechanism that the reduced LXRβ level by histone modification accounts for the etiology of chronic pain. In brief, (1) LXRβ deficits in ACC were involved in chronic inflammatory pain. (2) LXRβ knockdown by lentiviral shRNA in ACC led to hyperalgesia. (3) LXRβ activation by agonist GW exerted analgesic effect by inhibiting inflammation responses and enhanced excitatory neurotransmission in ACC. (4) Elevated HDAC5 expression was negatively correlated with that of LXRβ in ACC in chronic inflammatory pain. HDAC5 triggered histone deacetylation on the promoter region of *Lxrβ*, resulting in the downregulation of *Lxrβ* transcription. Therefore, our results might provide not only a better understanding of the role and regulatory mechanism of LXRβ in CIP but also a novel therapeutic target for chronic pain.

## Additional files

Additional file 1: **Table S1.** PCR primers and data analysis used in this study. (DOCX 50 kb)

Additional file 2: **Figure S1.** The expression of LXRα and LXRβ in mice ACC. The brain slices containing ACC were collected and applied to immunofluorescent staining. a Representative immunofluorescent images of ACC labeled with LXRα (green), LXRβ (red), and Hoechst 33258 (blue) in mice. LXRβ was widely expressed in ACC, while LXRα was scarely expressed. Scale bars = 100 μm. (TIF 1038 kb)

Additional file 3: **Figure S2.** The expression of LXRβ but not LXRα decreased in ACC after CFA paw injection. a Representative Western blot of LXRα levels in ACC on day 1, 3, 5, 7, and 14 after CFA injection. b The histogram showed summarized data of a normalized to an internal control and expressed as a relative value. c Representative Western blot of downregulated LXRβ levels in ACC on day 1, 3, 5, 7, and 14 after CFA injection. d The histogram showed summarized data of c normalized to an internal control and expressed as a relative value. Error bars represent SEM. *n* = 5, ^**^p < 0.01, ^***^p < 0.001 vs. Sham group. (TIF 483 kb)

Additional file 4: **Figure S3.** Microinjection of lentiviral shRNAs into ACC did not alter mice locomotor activities. a Representative traveling tracks in OFT showed no difference in the total distance in each group b, and the time spent in the center arena c in shNC-, shLXRα-, and shLXRβ-infected group. d shNC, shLXRα, and shLXRβ infection had no effect on retention time of mice on rotarod. Error bars represent SEM. *n* = 5 for each group. (TIF 611 kb)

Additional file 5: **Figure S4.** GW3965 reversed CFA-mediated TNF-α induction by LXR activation. ELISA analysis revealed GW (10 mg/kg) treatment inhibited TNF-α induction in a serum and b ACC after CFA injection. GW treatment increased the levels of c, d ApoE and e, f ABCA1 in serum and ACC. Error bars represent SEM. *n* = 5, ^*^p < 0.05, ^**^p < 0.01 vs. Sham group; ^#^*p* < 0.05, ^##^*p* < 0.01, ^###^*p* < 0.001 vs*.* CFA-injected group. (TIF 101 kb)

Additional file 6: **Figure S5.** The cellular pattern of LXRβ colocalization in mice ACC. The brain slices containing ACC were stained for a1–a4 β-tubulin III+LXRβ, b1–b4 GFAP+LXRβ, c1–c4 Iba-1+LXRβ, d1–d4 CAMK IIα+LXRβ, and e1–e4 GAD67+LXRβ. LXRβ colocalized mainly with glutamatergic neurons (CAMK IIα positive), moderately with GABAergic neurons (GAD67 positive), a small part in microglia (Iba-1 positive) and in astrocyte (GFAP positive) in ACC. β-tubulin III, GFAP, Iba-1, CAMK IIα, and GAD67 showed in green, LXRβ showed in red, and Hoechst in blue. Scale bars = 100 μm. (TIF 2467 kb)

Additional file 7: **Figure S6.** GW3965 protected ER stress in ACC of CIP mice. a GW reversed mRNA levels of ER stress markers, *CHOP* and *ATP4*, in ACC of CIP mice. Error bars represent SEM. *n* = 4, ^*^p < 0.05, ^**^p < 0.01 vs. Sham group; ^#^*p* < 0.05 vs. CFA-injected group. (TIF 62 kb)

## Data Availability

The datasets supporting the conclusions of this article are included within the article and its additional files. All material used in this manuscript will be made available to researchers subject to confidentiality.

## Abbreviations

ABCA1: ATP-binding cassette transporter
ACC: Anterior cingulate cortex
AMPA: α-Amino-3-hydroxy-5-methyl-4-isoxazolepropionic acid
CFA: Complete Freund’s adjuvant
ChIP: Chromatin immunoprecipitation
CIP: Chronic inflammatory pain
CNS: Central nervous system
Co-IP: Co-immunoprecipitation
ELISA: Enzyme-linked immunosorbent assay
EPSCs: Excitatory postsynaptic currents
ERK: Extracellular regulated protein kinases
HDAC5: Histone deacetylase 5
HDACi: HDAC inhibitor
IκBα: NF-kappa-B inhibitor alpha
JNK: c-Jun N-terminal kinase
LXRs: Liver X receptors
MAPKs: Mitogen-activated protein kinases
mEPSC: Miniature EPSC
NF-κB: Nuclear factor-κB
shNC: Negative shRNA for LXRs
shRNA: Short hairpin RNA
TNF-α: Tumor necrosis factor-α
