# Supplementary material for: Epigenetic suppression of liver X receptor β in anterior cingulate cortex by HDAC5 drives CFA-induced chronic inflammatory pain
Source: J Neuroinflammation. 2019 Jun 29;16:132. doi: 10.1186/s12974-019-1507-3 (PMC6599528; doi:10.1186/s12974-019-1507-3)
Supplement: Supplementary file 1 — Table S1. PCR primers and data analysis used in this study. (DOCX 50 kb) [file 12974_2019_1507_MOESM1_ESM.docx]

**Table 1**

| Gene | Primers | Primer sequence (5’-3’) |
| --- | --- | --- |
| LXRβ-I | -F | 5’-TGGTGATACTGAGAGGGACTGG-3’ |
|  | -R | 5’-CCAGCCACTAACCCATGCT-3’ |
| LXRβ-II | -F | 5’-ATGAGGCAGAGGCAGGAGGA -3’ |
|  | -R | 5’-ATGGTCTCGCCATTCTAGCTGT-3’ |
| LXRβ-III | -F | 5’-AAAGATTCCCAACTGTCCATAAGTG-3’ |
|  | -R | 5’-CTCAACCCCTCTCCCATAAAAG-3’ |
| LXRβ-IV | -F | 5’-ACCATCTTGCCATCTCCAAAT-3’ |
|  | -R | 5’-TGAGCCTGTTGCCTTTACCTC-3’ |
| LXRβ-total | -F | 5’-CTCTGCCTACATCGTGGTCATCT-3’ |
|  | -R | 5’-ATGAAGGCATCCATCTGGCAGGT-3’ |
| GAPDH | -F | 5’-TGTGTCCGTCGTGGATCTGA-3’ |
|  | -R | 5’-TTGCTGTTGAAGTCGCAGGAG-3’ |

| 1B, D  Sham (n = 6)  CFA (n = 6)  Sham+GW (n = 6)  CFA+GW (n = 6) | Two-Way ANOVA  LXRα  Animal model: F(1,24) = 0.104, p = 0.750  Drug treatment: F(1,24) = 0.457, p = 0.507  Animal model x Drug treatment: F(1,24) = 0.018, p = 0.895  LXRβ  Animal model: F(1,24) = 62.239, p < 0.001  Drug treatment: F(1,24) = 3.806, p = 0.065  Animal model x Drug treatment: F(1,24) = 0.335, p = 0.569 | *Post hoc* Tukey’s test:  Sham *vs*. CFA, p = 0.999  Sham+GW *vs*. CFA+GW, p = 0.988  Sham *vs.* Sham+GW, p = 0.980  CFA Veh *vs.* CFA+GW, p = 0.939  *Post hoc* Tukey’s test:  Sham *vs.* CFA, p < 0.001  Sham+GW *vs.* CFA+GW, p < 0.001  Sham *vs.* Sham+GW, p = 0.307  CFA Veh *vs.* CFA+GW, p = 0.768 |  |
| --- | --- | --- | --- |
| 1I  Sham (n = 6)  CFA (n = 6) | Unpaired Student’s t-test  T = 5.161, p = 0.004 |  | Sham 23.5 ± 2.81  CFA 13.0 ± 1.30 |
| 1J, K  shNC (n = 6)  shLXRα (n = 6)  shLXRβ (n = 6) | Unpaired Student’s t-test  LXRα  T = 2.000, p = 0.002  LXRβ  T = 1.584, p = 0.007 |  | LXRα  shNC 100 ± 3.20  shLXRα 52 ± 4.47  LXRβ  shNC 100 ± 8.82  shLXRα 43 ± 7.52 |
| 1L  shNC (n = 5)  shLXRα (n = 5)  shLXRβ (n = 5)  shLXRα+ shLXRβ (n = 5) | One-Way ANOVA  D8  F (3,16) = 4.092, p = 0.025  D9  F (3,16) = 4.247, p = 0.022  D11  F (3,16) = 1.913, p = 0.168  D13  F (3,16) = 0.886, p = 0.469  D15  F (3,16) = 2.400, p = 0.106  D22  F (3,16) = 2.507, p = 0.096 | *Post hoc* Dunnett T3 test:  shNC *vs.* shLXRα, p = 0.998  shNC *vs.* shLXRβ, p = 0.156  shNC *vs.* shLXRα +shLXRβ, p = 0.139  *Post hoc* Dunnett T3 test:  shNC *vs.* shLXRα, p = 0.992  shNC *vs.* shLXRβ, p = 0.233  shNC *vs.* shLXRα +shLXRβ, p = 0.148  *Post hoc* Dunnett T3 test:  shNC *vs.* shLXRα, p = 1.000  shNC *vs.* shLXRβ, p = 0.756  shNC *vs.* shLXRα +shLXRβ, p = 0.422  *Post hoc* LSD test:  shNC *vs.* shLXRα, p = 0.533  shNC *vs.* shLXRβ, p = 0.258  shNC *vs.* shLXRα +shLXRβ, p = 0.147  *Post hoc* Dunnett T3 test:  shNC *vs.* shLXRα, p = 1.000  shNC *vs.* shLXRβ, p = 0.711  shNC *vs.* shLXRα +shLXRβ, p = 0.399  *Post hoc* Dunnett T3 test:  shNC *vs.* shLXRα, p = 0.994  shNC *vs.* shLXRβ, p = 0.682  shNC *vs.* shLXRα +shLXRβ, p = 0.319 |  |
| 1M  shNC (n = 8)  shLXRα (n = 8)  shLXRβ (n = 7)  shLXRα+ shLXRβ (n = 8) | One-Way ANOVA  D8  F(3.27) = 7.264, p = 0.001  D9  F(3.27) = 16.220, p < 0.001  D11  F(3.27) = 18.258, p < 0.001  D13  F(3.27) = 22.209, p < 0.001  D15  F(3.27) = 16.690, p < 0.001  D22  F(3.27) = 23.604, p < 0.001 | *Post hoc* Dunnett T3 test:  shNC *vs.* shLXRα, p = 0.930  shNC *vs.* shLXRβ, p = 0.011  shNC *vs.* shLXRα +shLXRβ, p = 0.034  *Post hoc* Dunnett T3 test:  shNC *vs.* shLXRα, p = 0.780  shNC *vs.* shLXRβ, p < 0.001  shNC *vs.* shLXRα +shLXRβ, p = 0.005  *Post hoc* Dunnett T3 test:  shNC *vs.* shLXRα, p = 0.890  shNC *vs.* shLXRβ, p = 0.006  shNC *vs.* shLXRα +shLXRβ, p = 0.003  *Post hoc* Dunnett T3 test:  shNC *vs.* shLXRα, p = 0.223  shNC *vs.* shLXRβ, p = 0.042  shNC *vs.* shLXRα +shLXRβ, p = 0.001  *Post hoc* Dunnett T3 test:  shNC *vs.* shLXRα, p = 0.960  shNC *vs.* shLXRβ, p = 0.019  shNC *vs.* shLXRα +shLXRβ, p = 0.001  *Post hoc* Dunnett T3 test:  shNC *vs.* shLXRα, p = 0.996  shNC *vs.* shLXRβ, p < 0.001  shNC *vs.* shLXRα +shLXRβ, p = 0.001 |  |
| 2B  Sham (n = 5)  CFA (n = 5)  CFA+1mg/kg GW (n = 5)  CFA+10mg/kg GW (n = 5) | One-Way ANOVA  d0  F(3.16) = 0.251, p = 0.859,  d1  F(3.16) = 181.376, p < 0.001  d3  F(3.16) = 210.506, p < 0.001  d5  F(3.16) = 22.209, p < 0.001  d7  F(3.16) = 262.646, p < 0.001  d14  F(3.16) = 71.689, p < 0.001 | *Post hoc* Dunnett T3 test:  Sham *vs.* CFA, p = 0.999  Sham *vs.* CFA+1mg/kg GW, p = 0.897  Sham *vs.* CFA+10mg/kg GW, p = 1.000  CFA+1mg/kg GW *vs*. CFA, p = 0.993  CFA+10mg/kg GW *vs*. CFA, p =1.000  *Post hoc* LSD test:  Sham *vs.* CFA, p < 0.001  Sham *vs.* CFA+1mg/kg GW, p < 0.001  Sham *vs.* CFA+10mg/kg GW, p < 0.001  CFA+1mg/kg GW *vs*. CFA, p = 0.809  CFA+10mg/kg GW *vs*. CFA, p = 0.543  *Post hoc* LSD test:  Sham *vs.* CFA, p < 0.001  Sham *vs.* CFA+1mg/kg GW, p < 0.001  Sham *vs.* CFA+10mg/kg GW, p < 0.001  CFA+1mg/kg GW *vs*. CFA, p = 0.509  CFA+10mg/kg GW *vs*. CFA, p = 0.335  *Post hoc* LSD test:  Sham *vs.* CFA, p < 0.001  Sham *vs.* CFA+1mg/kg GW, p < 0.001  Sham *vs.* CFA+10mg/kg GW, p < 0.001  CFA+1mg/kg GW *vs*. CFA, p = 0.714  CFA+10mg/kg GW *vs*. CFA, p = 0.424  *Post hoc* LSD test:  Sham *vs.* CFA, p < 0.001  Sham *vs.* CFA+1mg/kg GW, p < 0.001  Sham *vs.* CFA+10mg/kg GW, p < 0.001  CFA+1mg/kg GW *vs*. CFA, p = 0.510  CFA+10mg/kg GW *vs*. CFA, p = 0.004  *Post hoc* LSD test:  Sham *vs.* CFA, p < 0.001  Sham *vs.* CFA+1mg/kg GW, p < 0.001  Sham *vs.* CFA+10mg/kg GW, p < 0.001  CFA+1mg/kg GW *vs*. CFA, p = 0.647  CFA+10mg/kg GW *vs*. CFA, p = 0.001 |  |
| 2D  Sham (n = 5)  CFA (n = 5)  CFA+1mg/kg GW (n = 5)  CFA+10mg/kg GW (n = 5) | One-Way ANOVA  d0  F(3.16) = 0.254, p = 0.858  d1  F(3.16) = 187.723, p < 0.001    d3  F(3.16) = 30.354, p < 0.001  d5  F(3.16) = 60.931, p < 0.001  d7  F(3.16) = 422.091, p < 0.001  d14  F(3.16) = 64.951, p < 0.001 | *Post hoc* LSD test:  Sham *vs.* CFA, p = 0.530  Sham *vs.* CFA+1mg/kg GW, p = 0.515  Sham *vs.* CFA+10mg/kg GW, p = 0.440  CFA+1mg/kg GW *vs*. CFA, p = 0.980  CFA+10mg/kg GW *vs*. CFA, p = 0.882  *Post hoc* Dunnett T3 test:  Sham *vs.* CFA, p < 0.001  Sham *vs.* CFA+1mg/kg GW, p < 0.001  Sham *vs.* CFA+10mg/kg GW, p < 0.001  CFA+1mg/kg GW *vs*. CFA, p = 0.085  CFA+10mg/kg GW *vs*. CFA, p < 0.001  *Post hoc* LSD test:  Sham *vs.* CFA, p < 0.001  Sham *vs.* CFA+1mg/kg GW, p < 0.001  Sham *vs.* CFA+10mg/kg GW, p < 0.001  CFA+1mg/kg GW *vs*. CFA, p = 0.527  CFA+10mg/kg GW *vs*. CFA, p = 0.045  *Post hoc* LSD test:  Sham *vs*. CFA, p < 0.001  Sham *vs.* CFA+1mg/kg GW, p < 0.001  Sham *vs.* CFA+10mg/kg GW, p < 0.001  CFA+1mg/kg GW *vs*. CFA, p = 0.252  CFA+10mg/kg GW *vs*. CFA, p = 0.013  *Post hoc* Dunnett T3 test:  Sham *vs.* CFA, p < 0.001  Sham *vs.* CFA+1mg/kg GW, p < 0.001  Sham *vs.* CFA+10mg/kg GW, p < 0.001  CFA+1mg/kg GW *vs*. CFA, p < 0.001  CFA+10mg/kg GW *vs*. CFA, p < 0.001  *Post hoc* Dunnett T3 test:  Sham *vs.* CFA, p = 0.001  Sham *vs.* CFA+1mg/kg GW, p = 0.004  Sham *vs.* CFA+10mg/kg GW, p = 0.004  CFA+1mg/kg GW *vs*. CFA, p < 0.001  CFA+10mg/kg GW *vs*. CFA, p = 0.001 |  |
| 3B  shNC (n = 5)  shNC + CFA (n = 5)  shLXRα+CFA (n = 5)  shNC+CFA+GW (n = 5)  shLXRα+CFA+GW (n = 5) | One-Way ANOVA  ipsilateral  d0  F (4.20) = 0.506, p = 0.732  d1  F (4.20) = 132.724, p < 0.001  d3  F (4.20) = 100.261, p < 0.001  d5  F (4.20) = 79.948, p < 0.001  d7  F (4.20) = 128.184, p < 0.001  d14  F (4.20) = 49.534, p < 0.001 | *Post hoc* LSD test:  shNC+CFA *vs*. shNC, p = 0.336  shLXRα+CFA *vs*. shNC+CFA, p = 0.240  shNC+CFA+GW *vs*. shNC +CFA, p = 0.533  shLXRα+CFA+GW *vs*. shNC+CFA, p = 0.254  shLXRα+CFA+GW *vs*. shLXRα+CFA, p = 0.971  *Post hoc* LSD test:  shNC+CFA *vs*. shNC, p < 0.001  shLXRα+CFA *vs*. shNC+CFA, p = 0.884  shNC+CFA+GW *vs*. shNC +CFA, p = 0.437  shLXRα+CFA+GW *vs*. shNC+CFA, p = 0.415  shLXRα+CFA+GW *vs*. shLXRα+CFA, p = 0.339  *Post hoc* LSD test:  shNC+CFA *vs*. shNC, p < 0.001  shLXRα+CFA *vs*. shNC+CFA, p = 0.916  shNC+CFA+GW *vs*. shNC +CFA, p = 0.530  shLXRα+CFA+GW *vs*. shNC+CFA, p = 0.515  shLXRα+CFA+GW *vs*. shLXRα+CFA, p = 0.450  *Post hoc* LSD test:  shNC+CFA *vs*. shNC, p < 0.001  shLXRα+CFA *vs*. shNC+CFA, p = 0.991  shNC+CFA+GW *vs*. shNC +CFA, p = 0.103  shLXRα+CFA+GW *vs*. shNC+CFA, p = 0.229  shLXRα+CFA+GW *vs*. shLXRα+CFA, p = 0.234  *Post hoc* LSD test:  shNC+CFA *vs*. shNC, p < 0.001  shLXRα+CFA *vs*. shNC+CFA, p = 0.924  shNC+CFA+GW *vs*. shNC +CFA, p = 0.034  shLXRα+CFA+GW *vs*. shNC+CFA, p = 0.018  shLXRα+CFA+GW *vs*. shLXRα+CFA, p = 0.015  *Post hoc* LSD test:  shNC+CFA *vs*. shNC, p < 0.001  shLXRα+CFA *vs*. shNC+CFA, p = 0.954  shNC+CFA+GW *vs*. shNC +CFA, p = 0.005  shLXRα+CFA+GW *vs*. shNC+CFA, p = 0.022  shLXRα+CFA+GW *vs*. shLXRα+CFA, p = 0.025 |  |
| 3D  shNC (n = 5)  shNC + CFA (n = 5)  shLXRβ+CFA (n = 5)  shNC+CFA+GW (n = 5)  shLXRβ+CFA+GW (n = 5) | One-Way ANOVA  ipsilateral  d0  F (4.20) = 2.392, p = 0.085  d1  F (4.20) = 136.928, p < 0.001  d3  F (4.20) = 102.166, p < 0.001  d5  F (4.20) = 77.244, p < 0.001  d7  F (4.20) = 125.185, p < 0.001  d14  F (4.20) = 53.421, p < 0.001 | *Post hoc* Dunnett T3 test:  shNC+CFA *vs*. shNC, p = 0.834  shLXRβ+CFA *vs*. shNC+CFA, p = 0.062  shNC+CFA+GW *vs*. shNC +CFA, p = 0.996  shLXRβ+CFA+GW *vs*. shNC+CFA, p = 0.691  shLXRβ+CFA+GW *vs*. shLXRβ+CFA, p = 1.000  *Post hoc* LSD test:  shNC+CFA *vs*. shNC, p < 0.001  shLXRβ+CFA *vs*. shNC+CFA, p = 0.860  shNC+CFA+GW *vs*. shNC +CFA, p = 0.435  shLXRβ+CFA+GW *vs*. shNC+CFA, p = 0.950  shLXRβ+CFA+GW *vs*. shLXRβ+CFA, p = 0.909  *Post hoc* LSD test:  shNC+CFA *vs*. shNC, p < 0.001  shLXRβ+CFA *vs*. shNC+CFA, p = 0.844  shNC+CFA+GW *vs*. shNC +CFA, p = 0.530  shLXRβ+CFA+GW *vs*. shNC+CFA, p = 0.926  shLXRβ+CFA+GW *vs*. shLXRβ+CFA, p = 0.772  *Post hoc* LSD test:  shNC+CFA *vs*. shNC, p < 0.001  shLXRβ+CFA *vs*. shNC+CFA, p = 0.796  shNC+CFA+GW *vs*. shNC +CFA, p = 0.107  shLXRβ+CFA+GW *vs*. shNC+CFA, p = 0.262  shLXRβ+CFA+GW *vs*. shLXRβ+CFA, p = 0.383  *Post hoc* LSD test:  shNC+CFA *vs*. shNC, p < 0.001  shLXRβ+CFA *vs*. shNC+CFA, p = 0.963  shNC+CFA+GW *vs*. shNC +CFA, p = 0.038  shLXRβ+CFA+GW *vs*. shNC+CFA, p = 0.270  shLXRβ+CFA+GW *vs*. shLXRβ+CFA, p = 0.290  *Post hoc* LSD test:  shNC+CFA *vs*. shNC, p < 0.001  shLXRβ+CFA *vs*. shNC+CFA, p = 0.954  shNC+CFA+GW *vs*. shNC +CFA, p = 0.005  shLXRβ+CFA+GW *vs*. shNC+CFA, p = 0.550  shLXRβ+CFA+GW *vs*. shLXRβ+CFA, p = 0.513 |  |
| 3F  shNC (n = 6)  shNC + CFA (n = 6)  shLXRα+CFA (n = 6)  shNC+CFA+GW (n = 6)  shLXRα+CFA+GW (n = 6) | One-Way ANOVA  ipsilateral  d0  F (4.25) = 2.547, p = 0.064  d1  F (4.25) = 161.400, p < 0.001  d3  F (4.25) = 124.937, p < 0.001  d5  F (4.25) = 59.261, p < 0.001  d7  F (4.25) = 35.623, p < 0.001  d14  F (4.25) = 59.261, p < 0.001 | *Post hoc* Dunnett T3 test:  shNC+CFA *vs*. shNC, p = 0.616  shLXRα+CFA *vs*. shNC+CFA, p = 0.090  shNC+CFA+GW *vs*. shNC +CFA, p = 1.000  shLXRα+CFA+GW *vs*. shNC+CFA, p = 0.141  shLXRα+CFA+GW *vs*. shLXRα+CFA, p = 1.000  *Post hoc* Dunnett T3 test:  shNC+CFA *vs*. shNC, p < 0.001  shLXRα+CFA *vs*. shNC+CFA, p = 0.140  shNC+CFA+GW *vs*. shNC +CFA, p = 0.007  shLXRα+CFA+GW *vs*. shNC+CFA, p < 0.001  shLXRα+CFA+GW *vs*. shLXRα+CFA, p < 0.001  *Post hoc* Dunnett T3 test:  shNC+CFA *vs*. shNC, p < 0.001  shLXRα+CFA *vs*. shNC+CFA, p = 0.849  shNC+CFA+GW *vs*. shNC +CFA, p = 0.017  shLXRα+CFA+GW *vs*. shNC+CFA, p = 0.222  shLXRα+CFA+GW *vs*. shLXRα+CFA, p = 0.986  *Post hoc* Dunnett T3 test:  shNC+CFA *vs*. shNC, p < 0.001  shLXRα+CFA *vs*. shNC+CFA, p = 0.961  shNC+CFA+GW *vs*. shNC +CFA, p = 0.706  shLXRα+CFA+GW *vs*. shNC+CFA, p = 0.677  shLXRα+CFA+GW *vs*. shLXRα+CFA, p = 0.247  *Post hoc* Dunnett T3 test:  shNC+CFA *vs*. shNC, p < 0.001  shLXRα+CFA *vs*. shNC+CFA, p = 0.929  shNC+CFA+GW *vs*. shNC +CFA, p = 0.002  shLXRα+CFA+GW *vs*. shNC+CFA, p = 0.002  shLXRα+CFA+GW *vs*. shLXRα+CFA, p = 0.045  *Post hoc* Dunnett T3 test:  shNC+CFA *vs*. shNC, p < 0.001  shLXRα+CFA *vs*. shNC+CFA, p = 1.000  shNC+CFA+GW *vs*. shNC +CFA, p = 0.013  shLXRα+CFA+GW *vs*. shNC+CFA, p = 0.005  shLXRα+CFA+GW *vs*. shLXRα+CFA, p = 0.007 |  |
| 3G  shNC (n = 6)  shNC + CFA (n = 6)  shLXRα+CFA (n = 6)  shNC+CFA+GW (n = 6)  shLXRα+CFA+GW (n = 6) | Contralateral  d0  F (4.25) = 0.671, p = 0.618  d1  F (4.25) = 1.375, p = 0.271  d3  F (4.25) = 3.940, p = 0.013  d5  F (4.25) = 1.088, p = 0.384  d7  F (4.25) = 1.156, p = 0.354  d14  F (4.25) = 2.030, p = 0.121 | *Post hoc* Dunnett T3 test:  shNC+CFA *vs*. shNC, p = 0.995  shLXRα+CFA *vs*. shNC+CFA, p = 0.805  shNC+CFA+GW *vs*. shNC +CFA, p = 0.979  shLXRα+CFA+GW *vs*. shNC+CFA, p = 0.681  shLXRα+CFA+GW *vs*. shLXRα+CFA, p = 1.000  *Post hoc* Dunnett T3 test:  shNC+CFA *vs*. shNC, p = 1.000  shLXRα+CFA *vs*. shNC+CFA, p = 1.000  shNC+CFA+GW *vs*. shNC +CFA, p = 1.000  shLXRα+CFA+GW *vs*. shNC+CFA, p = 0.617  shLXRα+CFA+GW *vs*. shLXRα+CFA, p = 0.640  *Post hoc* Dunnett T3 test:  shNC+CFA *vs*. shNC, p = 0.997  shLXRα+CFA *vs*. shNC+CFA, p = 0.232  shNC+CFA+GW *vs*. shNC +CFA, p = 0.391  shLXRα+CFA+GW *vs*. shNC+CFA, p = 0.077  shLXRα+CFA+GW *vs*. shLXRα+CFA, p = 0.847  *Post hoc* LSD test:  shNC+CFA *vs*. shNC, p = 0.958  shLXRα+CFA *vs*. shNC+CFA, p = 0.241  shNC+CFA+GW *vs*. shNC +CFA, p = 0.527  shLXRα+CFA+GW *vs*. shNC+CFA, p = 0.116  shLXRα+CFA+GW *vs*. shLXRα+CFA, p = 0.673  *Post hoc* Dunnett T3 test:  shNC+CFA *vs*. shNC, p = 0.992  shLXRα+CFA *vs*. shNC+CFA, p = 0.990  shNC+CFA+GW *vs*. shNC +CFA, p = 1.000  shLXRα+CFA+GW *vs*. shNC+CFA, p = 0.620  shLXRα+CFA+GW *vs*. shLXRα+CFA, p = 0.629  *Post hoc* Dunnett T3 test:  shNC+CFA *vs*. shNC, p = 0.716  shLXRα+CFA *vs*. shNC+CFA, p = 0.950  shNC+CFA+GW *vs*. shNC +CFA, p = 1.000  shLXRα+CFA+GW *vs*. shNC+CFA, p = 0.754  shLXRα+CFA+GW *vs*. shLXRα+CFA, p = 0.980 |  |
| 3H  shNC (n = 6)  shNC + CFA (n = 6)  shLXRβ+CFA (n = 6)  shNC+CFA+GW (n = 6)  shLXRβ+CFA+GW (n = 6) | One-Way ANOVA  ipsilateral  d0  F (4.25) = 16.602, p = < 0.001  d1  F (4.25) = 192.393, p < 0.001  d3  F (4.25) = 253.297, p < 0.001  d5  F (4.25) = 77.145, p < 0.001  d7  F (4.25) = 60.959, p < 0.001  d14  F (4.25) = 71.625, p < 0.001 | *Post hoc* Dunnett T3 test:  shNC+CFA *vs*. shNC, p = 1.000  shLXRβ+CFA *vs*. shNC+CFA, p = 0.001  shNC+CFA+GW *vs*. shNC +CFA, p = 1.000  shLXRβ+CFA+GW *vs*. shNC+CFA, p = 0.002  shLXRβ+CFA+GW *vs*. shLXRβ+CFA, p = 0.411  *Post hoc* LSD test:  shNC+CFA *vs*. shNC, p < 0.001  shLXRβ+CFA *vs*. shNC+CFA, p = 0.015  shNC+CFA+GW *vs*. shNC +CFA, p = 0.001  shLXRβ+CFA+GW *vs*. shNC+CFA, p = 0.342  shLXRβ+CFA+GW *vs*. shLXRβ+CFA, p = 0.122  *Post hoc* Dunnett T3 test:  shNC+CFA *vs*. shNC, p < 0.001  shLXRβ+CFA *vs*. shNC+CFA, p = 0.024  shNC+CFA+GW *vs*. shNC +CFA, p = 0.017  shLXRβ+CFA+GW *vs*. shNC+CFA, p = 0.073  shLXRβ+CFA+GW *vs*. shLXRβ+CFA, p = 0.988  *Post hoc* Dunnett T3 test:  shNC+CFA *vs*. shNC, p < 0.001  shLXRβ+CFA *vs*. shNC+CFA, p = 0.031  shNC+CFA+GW *vs*. shNC +CFA, p = 0.706  shLXRβ+CFA+GW *vs*. shNC+CFA, p = 0.785  shLXRβ+CFA+GW *vs*. shLXRβ+CFA, p = 0.435  *Post hoc* Dunnett T3 test:  shNC+CFA *vs*. shNC, p < 0.001  shLXRβ+CFA *vs*. shNC+CFA, p = 0.030  shNC+CFA+GW *vs*. shNC +CFA, p = 0.014  shLXRβ+CFA+GW *vs*. shNC+CFA, p = 0.751  shLXRβ+CFA+GW *vs*. shLXRβ+CFA, p = 0.508  *Post hoc* Dunnett T3 test:  shNC+CFA *vs*. shNC, p < 0.001  shLXRβ+CFA *vs*. shNC+CFA, p = 0.004  shNC+CFA+GW *vs*. shNC +CFA, p = 0.013  shLXRβ+CFA+GW *vs*. shNC+CFA, p = 0.011  shLXRβ+CFA+GW *vs*. shLXRβ+CFA, p = 0.990 |  |
| 3I  shNC (n = 6)  shNC + CFA (n = 6)  shLXRβ+CFA (n = 6)  shNC+CFA+GW (n = 6)  shLXRβ+CFA+GW (n = 6) | Contralateral  d0  F (4.25) = 14.545, p < 0.001  d1  F (4.25) = 19.595, p < 0.001  d3  F (4.25) = 29.381, p < 0.001  d5  F (4.25) = 17.005, p < 0.001  d7  F (4.25) = 31.770, p < 0.001  d14  F (4.25) = 42.599, p < 0.001 | *Post hoc* Dunnett T3 test:  shNC+CFA *vs*. shNC, p = 0.995  shLXRα+CFA *vs*. shNC+CFA, p = 0.001  shNC+CFA+GW *vs*. shNC +CFA, p = 0.979  shLXRα+CFA+GW *vs*. shNC+CFA, p = 0.003  shLXRα+CFA+GW *vs*. shLXRα+CFA, p = 0.987  *Post hoc* Dunnett T3 test:  shNC+CFA *vs*. shNC, p = 1.000  shLXRα+CFA *vs*. shNC+CFA, p = 0.003  shNC+CFA+GW *vs*. shNC +CFA, p = 1.000  shLXRα+CFA+GW *vs*. shNC+CFA, p = 0.045  shLXRα+CFA+GW *vs*. shLXRα+CFA, p = 0.070  *Post hoc* Dunnett T3 test:  shNC+CFA *vs*. shNC, p = 0.997  shLXRα+CFA *vs*. shNC+CFA, p < 0.001  shNC+CFA+GW *vs*. shNC +CFA, p = 0.391  shLXRα+CFA+GW *vs*. shNC+CFA, p = 0.001  shLXRα+CFA+GW *vs*. shLXRα+CFA, p = 0.809  *Post hoc* LSD test:  shNC+CFA *vs*. shNC, p = 0.956  shLXRα+CFA *vs*. shNC+CFA, p < 0.001  shNC+CFA+GW *vs*. shNC +CFA, p = 0.511  shLXRα+CFA+GW *vs*. shNC+CFA, p < 0.001  shLXRα+CFA+GW *vs*. shLXRα+CFA, p = 0.340  *Post hoc* Dunnett T3 test:  shNC+CFA *vs*. shNC, p = 0.992  shLXRα+CFA *vs*. shNC+CFA, p = 0.002  shNC+CFA+GW *vs*. shNC +CFA, p = 1.000  shLXRα+CFA+GW *vs*. shNC+CFA, p = 0.001  shLXRα+CFA+GW *vs*. shLXRα+CFA, p = 0.880  *Post hoc* Dunnett T3 test:  shNC+CFA *vs*. shNC, p = 0.716  shLXRα+CFA *vs*. shNC+CFA, p = < 0.001  shNC+CFA+GW *vs*. shNC +CFA, p = 1.000  shLXRα+CFA+GW *vs*. shNC+CFA, p = < 0.001  shLXRα+CFA+GW *vs*. shLXRα+CFA, p = 0.996 |  |
| 4D,E  Western blot  Sham (n = 5)  1d (n = 5)  3d (n = 5)  5d (n = 5) | One-Way ANOVA  Nucleus p65  F (4.20) = 26.726, p < 0.001  Cytoplasm p65  F (4.20) = 31.621, p < 0.001 | *Post hoc* Dunnett T3 test:  Sham *vs.* 1d, p < 0.001  Sham *vs.* 3d, p = 0.002  Sham *vs.* 5d, p < 0.001  Sham *vs.* 7d, p = 0.013  *Post hoc* Dunnett T3 test:  Sham *vs.* 1d, p = 0.039  Sham *vs*. 3d, p = 0.001  Sham *vs.* 5d, p < 0.001  Sham *vs.* 7d, p < 0.001 |  |
| 4F,G  Western blot  Sham (n = 5)  CFA (n = 5)  CFA+GW (n = 5) | One-Way ANOVA  Nucleus p65  F (2.12) = 24.045, p < 0.001  Cytoplasm p65  F (2.12) = 19.559, p < 0.001 | *Post hoc* Dunnett T3 test:  Sham *vs.* CFA, p < 0.001  CFA+GW *vs.* CFA, p = 0.027  *Post hoc* Dunnett T3 test:  Sham *vs.* CFA, p = 0.001  CFA+GW *vs.* CFA, p = 0.034 |  |
| 4H,I  Western blot  Sham (n = 5)  CFA (n = 5)  CFA+GW (n = 5) | One-Way ANOVA  Nucleus p50  F (2.12) = 50.237, p < 0.001  Cytoplasm p50  F (2.12) = 14.020, p = 0.001 | *Post hoc* Dunnett T3 test:  Sham *vs.* CFA, p < 0.001  CFA+GW *vs.* CFA, p = 0.002  *Post hoc* Dunnett T3 test:  Sham *vs.* CFA, p = 0.007  CFA+GW *vs.* CFA, p = 0.046 |  |
| 4J,K  Western blot  Sham (n = 5)  CFA (n = 5)  CFA+GW (n = 5) | One-Way ANOVA  p-IκBα  F (2.12) = 112.983, p < 0.001 | *Post hoc* Dunnett T3 test:  Sham *vs.* CFA, p < 0.001  CFA+GW *vs.* CFA, < 0.001 |  |
| 5G,H  Western blot  shNC+Sham (n = 4)  shLXRβ+Sham (n = 4)  shNC+CFA (n = 4)  shLXRβ+CFA (n = 4)  shNC+CFA+GW (n = 4)  shLXRβ+CFA+GW (n = 4) | One-Way ANOVA  p-ERK  F (5.18) = 28.418, p < 0.001 | *Post hoc* Dunnett T3 test:  shNC+CFA *vs.* shNC+Sham, p = 0.025  shLXRβ+CFA *vs.* shLXRβ+Sham, p = 0.001  shNC CFA+GW *vs.* shNC+CFA, p = 0.004  shLXRβ+CFA+GW *vs.* shLXRβ+CFA, p = 0.158  shLXRβ+CFA+GW *vs.* shNC+CFA+GW, p = 0.036 |  |
| 5I,J  Western blot  shNC+Sham (n = 4)  shLXRβ+Sham (n = 4)  shNC+CFA (n = 4)  shLXRβ+CFA (n = 4)  shNC+CFA+GW (n = 4)  shLXRβ+CFA+GW (n = 4) | One-Way ANOVA  p-JNK  F (5.18) = 25.838, p < 0.001 | *Post hoc* Dunnett T3 test:  shNC+CFA *vs.* shNC+Sham, p = 0.016  shLXRβ+CFA *vs.* shLXRβ+Sham, p = 0.026  shNC+CFA+GW *vs.* shNC+CFA, p = 0.002  shLXRβ+CFA+GW *vs.* shLXRβ+CFA, p = 0.074  shLXRβ+CFA+GW *vs.* shNC+CFA+GW, p = 0.239 |  |
| 5K,L  Western blot  shNC+Sham (n = 4)  shLXRβ+Sham (n=4)  shNC+CFA (n = 4)  shLXRβ+CFA (n = 4)  shNC+CFA+GW (n = 4)  shLXRβ+CFA+GW (n = 4) | One-Way ANOVA  p-p38  F (5.18) = 22.666, p < 0.001 | *Post hoc* Dunnett T3 test:  shNC+CFA *vs.* shNC+Sham, p = 0.800  shLXRβ+CFA *vs.* shLXRβ+Sham, p = 0.360  shNC+CFA+GW *vs.* shNC+CFA, p = 0.023  shLXRβ+CFA+GW *vs.* shLXRβ+CFA, p = 0.956  shLXRβ+CFA+GW *vs.* shNC CFA+GW, p = 0.007 |  |
| 6B  Sham (n = 6)  CFA+Veh (n = 6)  CFA+GW (n = 6) | One-Way ANOVA  10mV  F (2.15) = 8.631, p = 0.003  15mV  F (2.15) = 9.520, p = 0.002  20mV  F (2.15) = 9.781, p = 0.002  25mV  F (2.15) = 27.970, p < 0.001  30mV  F (2.15) = 25.407, p < 0.001  35mV  F (2.15) = 37.924, p < 0.001 | *Post hoc* Dunnett T3 test:  CFA *vs.* Saline, p = 0.006  CFA+GW *vs.* CFA, p = 0.225  *Post hoc* Dunnett T3 test:  CFA *vs.* Saline, p = 0.009  CFA+GW *vs.* CFA, p = 0.031  *Post hoc* Dunnett T3 test:  CFA *vs.* Saline, p = 0.006  CFA+GW *vs.* CFA, p = 0.050  *Post hoc* Dunnett T3 test:  CFA *vs.* Saline, p < 0.001  CFA+GW *vs.* CFA, p = 0.001  *Post hoc* Dunnett T3 test:  CFA *vs.* Saline, p < 0.001  CFA+GW *vs.* CFA, p = 0.002  *Post hoc* Dunnett T3 test:  CFA *vs.* Saline, p < 0.001  CFA+GW *vs.* CFA, p = 0.001 |  |
| 6E  Sham (n = 7 from 2 mice)  CFA+Veh (n= 9 from 3 mice)  CFA+GW (n= 9 from 3 mice) | One-Way ANOVA  Frequency  F (2.22) = 9.161, p = 0.001  Amplitude  F (2.22) = 0.452, p = 0.642 | *Post hoc* Dunnett T3 test:  CFA *vs.* Saline, p = 0.007  CFA+GW *vs.* CFA, p = 0.018  *Post hoc* Dunnett T3 test:  CFA *vs.* Saline, p = 0.829  CFA+GW *vs.* CFA, p = 0.788 |  |
| 6F,G,H  Sham (n=8)  CFA (n=8)  CFA+GW (n=8) | One-Way ANOVA  S831  F (2.21) = 11.642, p < 0.001  S845  F (2.21) = 10.334, p = 0.001 | *Post hoc* LSD test:  CFA *vs.* Saline, p < 0.001  CFA+GW *vs.* CFA, p = 0.002  *Post hoc* Dunnett T3 test:  CFA *vs.* Saline, p = 0.004  CFA+GW *vs.* CFA, p = 0.004 |  |
| 7A,B  Western blot  Sham (n = 4)  d1 (n = 4)  d3 (n = 4)  d5 (n = 4)  d7 (n = 4)  d14 (n = 4) | One-Way ANOVA  HDAC5  F (5.18) = 22.008, p < 0.001  HDAC2  F (5,18) =2.139, p = 0.107  LXRβ  F (5,18) = 4.782, p = 0.006 | *Post hoc* LSD test:  d1 *vs.* Sham, p < 0.001  d3 *vs.* Sham, p < 0.001  d5 *vs.* Sham, p < 0.001  d7 *vs.* Sham, p < 0.001  d14 *vs.* Sham, p = 0.252  *Post hoc* Dunnett T3 test:  d1 *vs.* Sham, p = 0.951  d3 *vs.* Sham, p = 0.999  d5 *vs.* Sham, p = 0.880  d7 *vs.* Sham, p = 0.937  d14 *vs.* Sham, p = 0.916  *Post hoc* LSD test:  Sham *vs.* 1d, p < 0.001  Sham *vs.* 3d, p = 0.002  Sham *vs.* 5d, p = 0.015  Sham *vs.* 7d, p = 0.036  Sham *vs.* 14d, p = 0.005 | Sham 100 ± 13.60  1d 206 ± 14.54  3d 204 ± 6.92  5d 200 ± 7.52  7d 233 ± 10.16  14d 129 ± 13.60  Sham 100 ± 4.69  1d 90 ± 3.61  3d 97 ± 1.52  5d 118 ± 7.34  7d 109 ± 2.28  14d 123 ± 6.76  Sham 100 ± 12.91  1d 51 ± 5.61  3d 63 ± 7.55  5d 72 ± 4.50  7d 76 ± 3.61  14d 66 ± 6.97 |
| 7C  DMSO (n = 10)  1h (n = 10)  3h (n = 10)  6h (n = 10)  24h (n = 10) | One-Way ANOVA  F (4,45) = 44.621, p < 0.001 | *Post hoc* LSD test  DMSO *vs.* 1h, p = 0.048  DMSO *vs.* 3h, p < 0.001  DMSO *vs.* 6h, p < 0.001  DMSO *vs.* 24h, p < 0.001 | 1h 3.3 ± 0.28  3h 8.2 ± 0.62  6h 13.5 ± 0.80  24h 12.2 ± 1.59 |
| 7D,E  Western blot  DMSO (n = 5)  1h (n = 5)  3h (n = 5)  6h (n = 5)  24h (n = 5) | One-Way ANOVA  AcH3  F (4,20) = 37.297, p < 0.001  AcH4  F (4,20) = 18.008, p < 0.001 | *Post hoc* Dunnett T3 test  DMSO *vs.* 1h, p < 0.001  DMSO *vs.* 3h, p = 0.004  DMSO *vs.* 6h, p = 0.001  DMSO *vs.* 24h, p < 0.001  *Post hoc* Dunnett T3 test  DMSO *vs.* 1h, p = 0.032  DMSO *vs.* 3h, p = 0.013  DMSO *vs.* 6h, p = 0.004  DMSO *vs.* 24h, p = 0.001 | DMSO 1.0 ± 0.05  1h 1.5 ± 0.05  3h 1.9 ± 0.12  6h 2.2 ± 0.13  24h 2.4 ± 0.12  DMSO 1.0 ± 0.08  1h 1.7 ± 0.17  3h 1.7 ± 0.13  6h 2.5 ± 0.20  24h 2.2 ± 0.13 |
| 8B  pI (n = 6)  pII (n = 5)  pIII ( n = 6)  pIV (n = 5)  IgG (n = 6) | One-Way ANOVA  AcH3  F (4,23) = 30.945, p < 0.001 | *Post hoc* LSD test  pI *vs.* IgG, p < 0.001  pII *vs.* IgG, p < 0.001  pIII *vs.* IgG, p = 0.799  pIV *vs.* IgG, p < 0.001 | pI 0.35 ± 0.02  pII 0.26 ± 0.05  pIII 0.03 ± 0.01  pIV 0.32 ± 0.04  IgG 0.02 ± 0.01  Beads 0.008 ± 0.004 |
| 8C  pI (n = 6)  pII (n = 6)  pIII (n = 6)  pIV (n = 6)  IgG (n = 6) | One-Way ANOVA  AcH4  F (4,25) = 23.761, p < 0.001 | *Post hoc* LSD test  pI *vs.* IgG, p < 0.001  pII *vs.* IgG, p < 0.001  pIII *vs.* IgG, p = 0.920  pIV *vs.* IgG, p < 0.001 | pI 5.0 ± 0.80  pII 7.3 ± 1.06  pIII 0.6 ± 0.11  pIV 7.1 ± 1.04  IgG 0.5 ± 0.15  Beads 0.2 ± 0.23 |
| 8D  DMSO  1h (n = 6)  3h (n = 3)  24h (n = 3)  SAHA  1h (n = 6)  3h (n = 5)  24h (n = 6)  DMSO  1h n = 6;  3h n = 3;  24h n = 3  SAHA  1h n = 5;  3h n = 5  24h n = 5 | Unpaired Student’s t-test  AcH3  1h T = 3.498, p < 0.001  3h T = 2.823, p = 0.013  24h T = 1.374, p = 0.337  AcH4  1h T = 0.635, p = 0.443  3h T = 1.374, p = 0.153  24h T = 0.371, p = 0.544 |  | 1h  DMSO 1.0 ± 0.06  SAHA 2.0 ± 0.10  3h  DMSO 1.0 ± 0.12  SAHA 2.3 ± 0.35  24h  DMSO 1.0 ± 0.27  SAHA 1.4 ± 0.36  1h  DMSO 1.0 ± 0.16  SAHA 1.3 ± 0.31  3h  DMSO 1.0 ± 0.11  SAHA 1.2 ± 0.05  24h  DMSO 1.0 ± 0.14  SAHA 1.1± 0.12 |
| 8E  DMSO  1h (n = 5)  3h (n = 3)  24h (n = 3)  SAHA  1h (n = 5)  3h (n = 5)  24h (n = 5)  DMSO  1h (n = 6)  3h (n = 3)  24h (n = 3)  SAHA  1h (n = 6)  3h (n = 5)  24h (n = 5) | Unpaired Student’s t-test  AcH3  1h T = 1.175, p < 0.001  3h T = 1.303, p = 0.005  24h T = 1.612, p = 0.202    AcH4  1h T = 7.982, p = 0.113  3h T = 0.175, p = 0.716  24h T = 0.618, p = 0.452 |  | 1h  DMSO 1.0 ± 0.20  SAHA 3.7 ± 0.39  3h  DMSO 1.0 ± 0.14  SAHA 2.3 ± 0.30  24h  DMSO 1.0 ± 0.21  SAHA 1.4 ± 0.21  1h  DMSO 1.0 ± 0.15  SAHA 1.3 ± 0.06  3h  DMSO 1.0 ± 0.27  SAHA 1.1 ± 0.22  24h  DMSO 1.0 ± 0.31  SAHA 1.3 ± 0.30 |
| 8F  DMSO  1h (n = 5)  3h (n = 3)  24h (n = 3)  SAHA  1h (n = 5)  3h (n = 5)  24h (n = 6)  DMSO  1h (n = 5)  3h (n = 3)  24h (n = 3)  SAHA  1h (n = 5)  3h (n = 5)  24h (n = 6) | Unpaired Student’s t-test  AcH3  1h T = 13.101, p = 0.007  3h T = 3.239, p < 0.001  24h T = 0.972, p = 0.405  AcH4  1h T = 11.410, p = 0.155  3h T = 2.114, p = 0.524  24h T = 0.110, p = 0.596 |  | 1h  DMSO 1.0 ± 0.16  SAHA 5.8 ± 0.32  3h  DMSO 1.0 ± 0.10  SAHA 3.1 ± 0.25  24h  DMSO 1.0 ± 0.21  SAHA 1.4 ± 0.40  1h  DMSO 1.0 ± 0.15  SAHA 1.2 ± 0.04  3h  DMSO 1.0 ± 0.14  SAHA 1.2 ± 0.23  24h  DMSO 1.0 ± 0.22  SAHA 1.1 ± 0.17 |
| Supplementary 2C,D  Sham (n = 5)  d1 (n = 5)  d3 (n = 5)  d5 (n = 5)  d7 (n = 5)  d14 (n = 5) | Unpaired Student’s t-test  LXRβ  d1, T = 9.151, p < 0.001  d3, T = 7.036, p < 0.001  d5, T = 5.768, p = 0.001  d7, T = 3.750, p = 0.008  d14, T = 3.980, p = 0.006 |  |  |
| Supplementary 3B  Open field  shNC (n =5)  shLXRα (n =5)  shLXRβ (n =5) | One-Way ANOVA  F (2,12) = 0.077, p = 0.926 | *Post hoc* Dunnett T3 test  shLXRα *vs.* shNC, p = 0.981  shLXRβ *vs.* shNC, p = 0.982 |  |
| Supplementary 3C  Open field  shNC (n =5)  shLXRα (n =5)  shLXRβ (n =5) | One-Way ANOVA  F (2,12) = 0.614, p = 0.557 | *Post hoc* Dunnett T3 test  shLXRα *vs.* shNC, p = 0.999  shLXRβ *vs.* shNC, p = 0.533 |  |
| Supplementary 3D  Rotarod test  shNC (n = 5)  shLXRα (n = 5)  shLXRβ (n = 5) | One-Way ANOVA  F (2,12) = 0.191, p = 0.829 | *Post hoc* Dunnett T3 test  shLXRα *vs.* shNC, p = 0.998  shLXRβ *vs.* shNC, p = 0.945 |  |
| Supplementary 4A  CFA (n = 5)  CFA+GW (n = 5) | One-Way ANOVA  Serum TNFα  F (4,20) = 20.676, p < 0.001  Unpaired Student’s t-test  d0 CFA+GW *vs.* CFA, T = 0.424, p = 0.688  d1 CFA+GW *vs.* CFA, T = 9.231, p < 0.001  d3 CFA+GW *vs.* CFA, T = 4.512, p = 0.003  d5 CFA+GW *vs.* CFA, T = 1.736, p = 0.121  d7 CFA+GW *vs.* CFA, T = -0.362, p = 0.732 | *Post hoc* Dunnett T3 test  d1 *vs.* d0, p = 0.030  d3 *vs.* d0, p = 0.025  d5 *vs.* d0, p = 0.252  d7 *vs.* d0, p = 0.997 |  |
| Supplementary 4B  CFA (n = 5)  CFA+GW (n = 5) | One-Way ANOVA  ACC TNFα  F (4,20) = 20.844, p < 0.001  Unpaired Student’s t-test  d0 CFA+GW *vs.* CFA, T = -0.169, p = 0.872  d1 CFA+GW *vs.* CFA, T =11.127, p < 0.001  d3 CFA+GW *vs.* CFA, T = 2.603, p = 0.049  d5 CFA+GW *vs.* CFA, T = 4.405, p = 0.003  d7 CFA+GW *vs.* CFA, T = 1.942, p = 0.109 | *Post hoc* Dunnett T3 test  d1 *vs.* d0, p = 0.001  d3 *vs.* d0, p = 0.006  d5 *vs.* d0, p = 0.003  d7 *vs.* d0, p = 0.162 |  |
| Supplementary 4C  CFA (n = 5)  CFA+GW (n = 5) | One-Way ANOVA  Serum ApoE  F (4,20) = 0.675, p = 0.625  Unpaired Student’s t-test  d0 CFA+GW *vs.* CFA, T =-2.259, p = 0.127  d1 CFA+GW *vs.* CFA, T = -3.191, p = 0.034  d3 CFA+GW *vs.* CFA, T = -6.277, p = 0.012  d5 CFA+GW *vs.* CFA, T = -3.527, p = 0.024  d7 CFA+GW *vs.* CFA, T = -4.425, p = 0.013 | *Post hoc* Dunnett T3 test  d1 *vs.* d0, p = 0.994  d3 *vs.* d0, p = 0.987  d5 *vs.* d0, p = 0.757  d7 *vs.* d0, p = 0.944 |  |
| Supplementary 4D  CFA (n = 5)  CFA+GW (n = 5) | One-Way ANOVA  ACC ApoE  F (4,20) = 0.484, p = 0.747  Unpaired Student’s t-test  d0 CFA+GW *vs.* CFA, T = -1.602, p = 0.186  d1 CFA+GW *vs.* CFA, T = -9.965, p = 0.001  d3 CFA+GW *vs.* CFA, T = -3.080, p = 0.049  d5 CFA+GW *vs.* CFA, T = -3.224, p = 0.032  d7 CFA+GW *vs.* CFA, T = -6.570, p = 0.008 | *Post hoc* Dunnett T3 test  d1 *vs.* d0, p = 0.692  d3 *vs.* d0, p = 1.000  d5 *vs.* d0, p = 1.000  d7 *vs.* d0, p = 1.000 |  |
| Supplementary 4E  CFA (n = 5)  CFA+GW (n = 5) | One-Way ANOVA  Serum ABCA1  F (4,20) = 3.517, p = 0.025  Unpaired Student’s t-test  d0 CFA+GW *vs.* CFA, T =2.062, p = 0.084  d1 CFA+GW *vs.* CFA, T = 1.451, p = 0.193  d3 CFA+GW *vs.* CFA, T = -0.140, p = 0.892  d5 CFA+GW *vs.* CFA, T = -4.062, p = 0.004  d7 CFA+GW *vs.* CFA, T = -2.509, p = 0.040 | *Post hoc* Dunnett T3 test  d1 *vs.* d0, p = 0.174  d3 *vs.* d0, p = 0.437  d5 *vs.* d0, p = 1.000  d7 *vs.* d0, p = 0.994 |  |
| Supplementary 4F  CFA (n = 5)  CFA+GW (n = 5) | One-Way ANOVA  ACC ABCA1  F (4,20) = 4.006, p = 0.015  Unpaired Student’s t-test  d0 CFA+GW *vs.* CFA, T = 1.186, p = 0279  d1 CFA+GW *vs.* CFA, T = -7.232, p < 0.001  d3 CFA+GW *vs.* CFA, T = -1.213, p = 0.276  d5 CFA+GW *vs.* CFA, T = -7.249, p < 0.001  d7 CFA+GW *vs.* CFA, T = -0.728, p < 0.001 | *Post hoc* Dunnett T3 test  d1 *vs.* d0, p = 0.512  d3 *vs.* d0, p = 0.925  d5 *vs.* d0, p = 0.533  d7 *vs.* d0, p = 1.000 |  |
| Supplementary 6A  Sham (n = 4)  GW (n = 4)  CFA (n = 4)  CFA+GW (n = 4) | One-Way ANOVA  *CHOP*  F (3,12) = 6.532, p = 0.012  *ATP4*  F (3,12) = 11.389, p = 0.001  *sXBP1*  F (3,12) = 0.603, p = 0.625  *tXBP1*  F (3,12) = 1.602, p = 0.256 | *Post hoc* LSD test  CFA *vs.* Sham, p = 0.003  CFA+GW *vs.* CFA, p = 0.013  *Post hoc* Dunnett T3 test  CFA *vs.* Sham, p = 0.032  CFA+GW *vs.* CFA, p = 0.039  *Post hoc* Dunnett T3 test  CFA *vs.* Sham, p = 0.479  CFA+GW *vs.* CFA, p = 0.957  *Post hoc* LSD test  CFA *vs.* Sham, p = 0.153  CFA+GW *vs.* CFA, p = 0.903 |  |
