# Supplementary figures and images for: Epigenetic suppression of liver X receptor β in anterior cingulate cortex by HDAC5 drives CFA-induced chronic inflammatory pain
Source: J Neuroinflammation. 2019 Jun 29;16:132. doi: 10.1186/s12974-019-1507-3 (PMC6599528; doi:10.1186/s12974-019-1507-3)

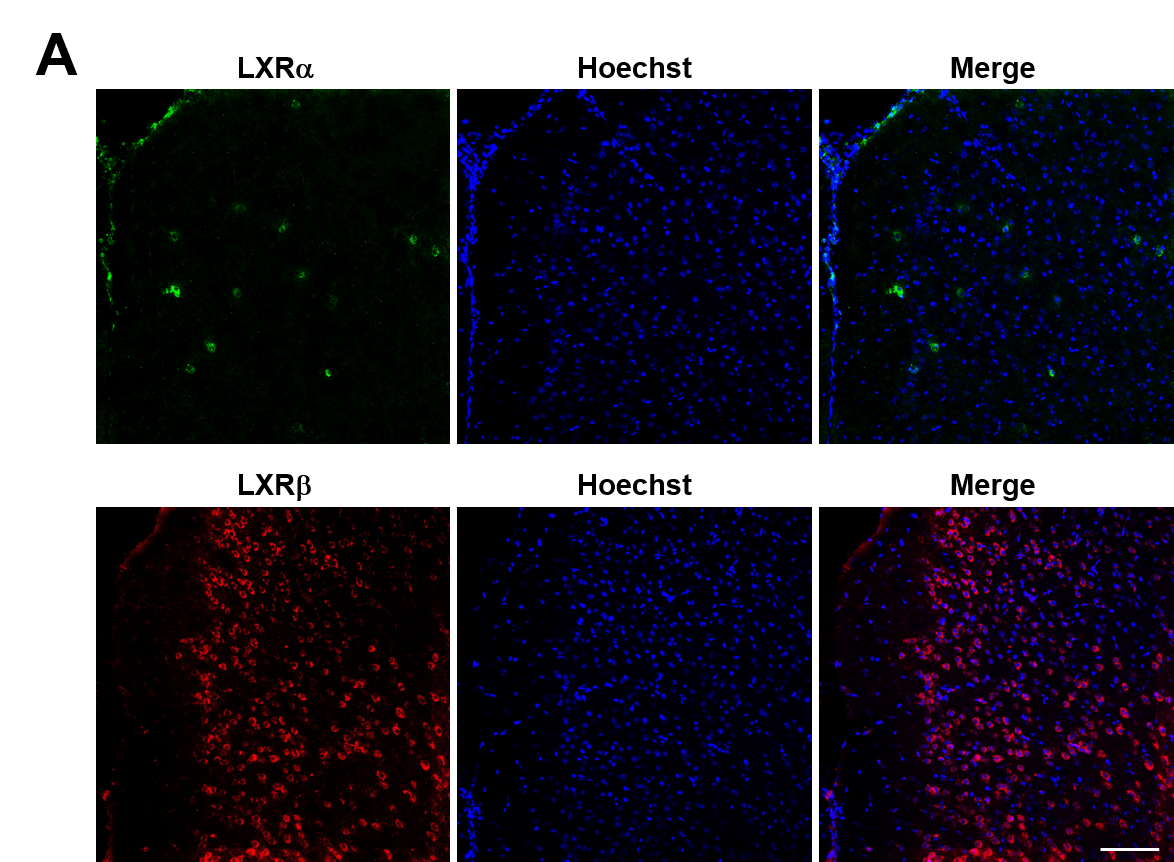

Supplement: Supplementary file 2 — Figure S1. The expression of LXRα and LXRβ in mice ACC. The brain slices containing ACC were collected and applied to immunofluorescent staining. a Representative immunofluorescent images of ACC labeled with LXRα (green), LXRβ (red), and Hoechst 33258 (blue) in mice. LXRβ was widely expressed in ACC, while LXRα was scarely expressed. Scale bars = 100 μm. (TIF 1038 kb) [file 12974_2019_1507_MOESM2_ESM.tif]

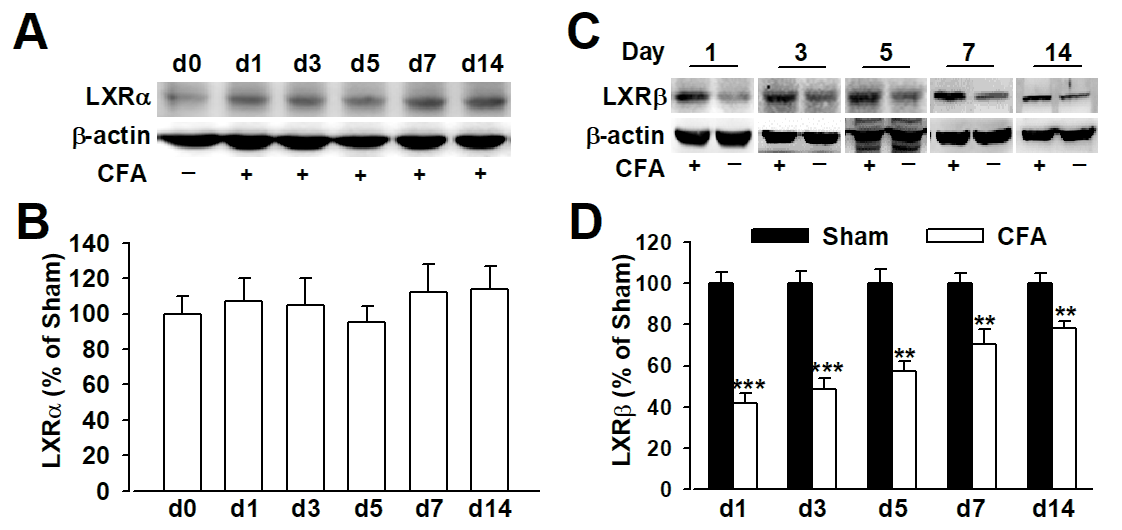

Supplement: Supplementary file 3 — Figure S2. The expression of LXRβ but not LXRα decreased in ACC after CFA paw injection. a Representative Western blot of LXRα levels in ACC on day 1, 3, 5, 7, and 14 after CFA injection. b The histogram showed summarized data of a normalized to an internal control and expressed as a relative value. c Representative Western blot of downregulated LXRβ levels in ACC on day 1, 3, 5, 7, and 14 after CFA injection. d The histogram showed summarized data of c normalized to an internal control and expressed as a relative value. Error bars represent SEM. n = 5, **p < 0.01, ***p < 0.001 vs. Sham group. (TIF 483 kb) [file 12974_2019_1507_MOESM3_ESM.tif]

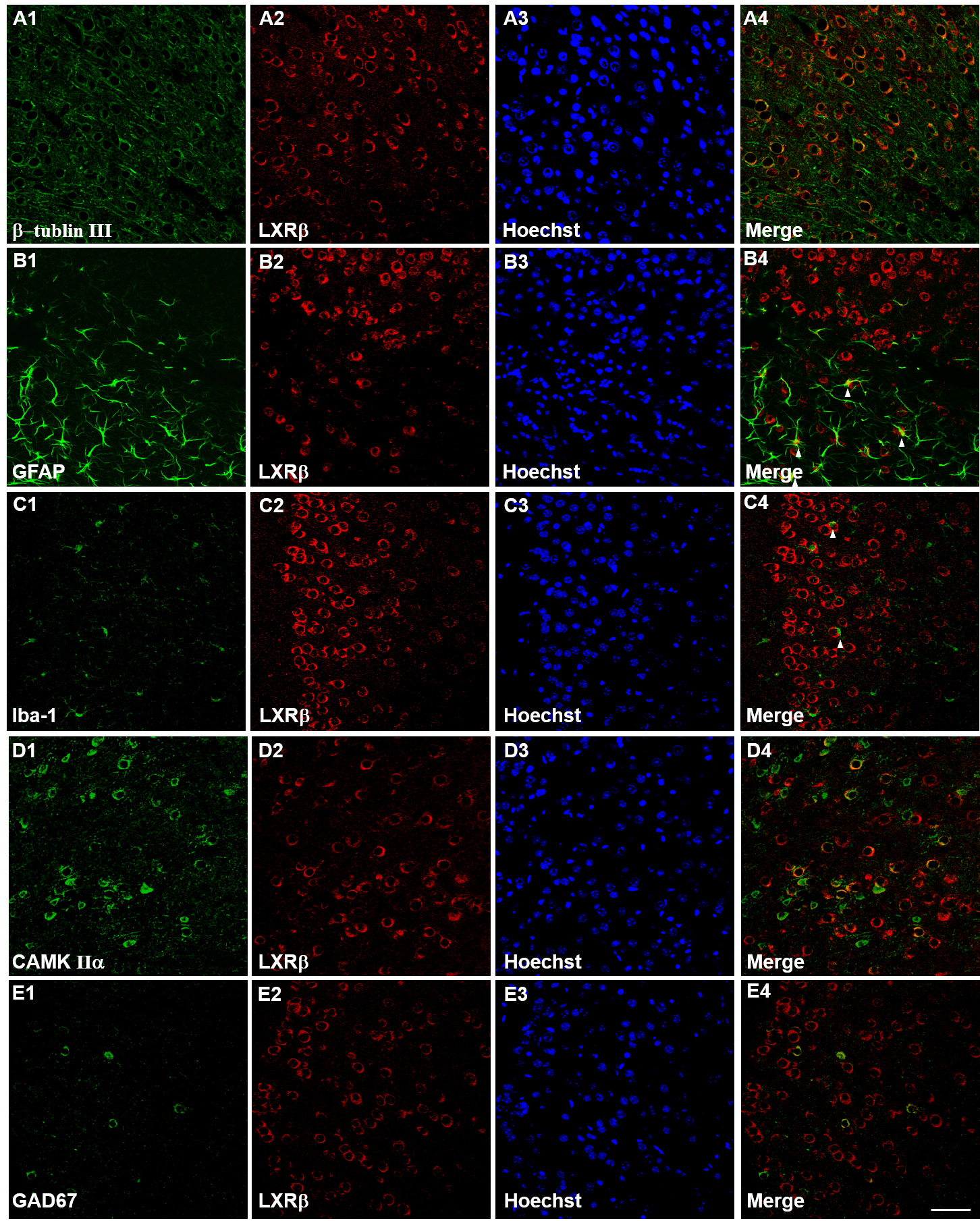

Supplement: Supplementary file 6 — Figure S5. The cellular pattern of LXRβ colocalization in mice ACC. The brain slices containing ACC were stained for a1–a4 β-tubulin III+LXRβ, b1–b4 GFAP+LXRβ, c1–c4 Iba-1+LXRβ, d1–d4 CAMK IIα+LXRβ, and e1–e4 GAD67+LXRβ. LXRβ colocalized mainly with glutamatergic neurons (CAMK IIα positive), moderately with GABAergic neurons (GAD67 positive), a small part in microglia (Iba-1 positive) and in astrocyte (GFAP positive) in ACC. β-tubulin III, GFAP, Iba-1, CAMK IIα, and GAD67 showed in green, LXRβ showed in red, and Hoechst in blue. Scale bars = 100 μm. (TIF 2467 kb) [file 12974_2019_1507_MOESM6_ESM.tif]
